# Simultaneous Feature Selection for Optimal Dynamic Treatment Regimens

**DOI:** 10.1002/sim.70169

**Published:** 2025-07-15

**Authors:** Mochuan Liu, Yuanjia Wang, Donglin Zeng

**Affiliations:** ^1^ Department of Biostatistics University of North Carolina at Chapel Hill Chapel Hill North Carolina USA; ^2^ Department of Biostatistics Columbia University New York New York USA; ^3^ Department of Biostatistics University of Michigan Ann Arbor Michigan USA

**Keywords:** decision‐making, dynamic treatment regimens, group lasso, ramp loss function, variable selection

## Abstract

Dynamic treatment regimens (DTRs), where treatment decisions are tailored to individual patient's characteristics and evolving health status over multiple stages, have gained increasing interest in the modern era of precision medicine. Identifying important features that drive these decisions over stages not only leads to parsimonious DTRs for practical use but also enhances the reliability of learning optimal DTRs. Existing methods for learning optimal DTRs, such as Q‐learning and O‐learning, rely on a sequential procedure to estimate the optimal decision at each stage backward. Incorporating feature selection in these methods through regularization at each stage of estimation only identifies unimportant tailoring variables at each stage but is not necessary for those variables that are not important across all the stages. As a result, false discovery errors are likely to accumulate over stages in these sequential methods. To overcome this limitation, we propose a framework, namely L1 multistage ramp loss (L1‐MRL) learning, to learn the optimal decision rules and, at the same time, perform variable selection across all the stages simultaneously. This framework uses a single multistage ramp loss to estimate optimal DTRs for all stages. Furthermore, a group Lasso‐type penalty is imposed to penalize the coefficients in the decision rules across all stages, which enables the identification of features that are important for at least one stage decision. Theoretically, we show that the estimator is consistent and enjoys the oracle property toward the optimal. We demonstrate that the proposed method performs equally well as or better than many existing DTR methods with variable selection capability via extensive simulation studies and an application to electronic health record (EHR) data for type 2 diabetes (T2D) patients.

## 1 Introduction

The dynamic treatment regimen is one of the most important strategies in precision medicine, where treatment decisions are tailored to an individual's characteristics and evolving health status over multiple stages [1]. Over the past two decades, many statistical and machine learning methods have been developed to learn the optimal DTRs. These include regression‐based approaches, such as A‐learning [2], G‐estimation [3], Q‐learning [4], weighed ordinary least squares [5], and regret regression [6, 7], which fit a series of regression models to estimate the expected reward or varied functions under any given treatment and then determine the optimal decision function that yields the highest reward outcome. More recently, value‐based approaches have been advocated, which directly maximize a value function within a class of treatment decision functions. These methods usually solve a weighted classification problem, such as O‐learning [8, 9, 10, 11, 12], or its doubly robust variants [13].

Identifying the key factors driving treatment decisions is also crucial for learning DTRs in clinical practice [14]. This not only leads to parsimonious DTRs but also aids in identifying less relevant features, enhancing the reliability of learning optimal DTRs, particularly given that many studies have limited sample sizes. Most methods developed for this purpose simply incorporate variable selection into the estimation steps of the existing learning algorithms. For example, in the regression methods [4, 15, 16, 17, 18, 19], variable selection is performed by imposing penalties on the parameters corresponding to the coefficients of each factor in the treatment decision stagewise. Similar considerations have been adopted for value‐based methods [20, 21, 22, 23, 24]. One major limitation of these approaches is that incorporating feature selection through regularization at each stage of estimation can only identify unimportant tailoring variables for each stage separately, rather than those variables that may not be important across all stages. Additionally, false discovery errors are likely to accumulate over stages in these sequential methods.

In this work, we propose a machine learning‐based approach to learn optimal DTRs with simultaneous variable selection capability across all stages to overcome this limitation. Specifically, we extend the multistage ramp loss (MRL) framework proposed by Liu et al. [25] by incorporating an additional group Lasso‐type penalty term to enforce sparsity over the decision rules. We name this new method L1‐MRL. The proposed method inherits the simultaneous estimation property of MRL, which unifies the estimation of treatment rules into a single optimization problem. This allows for cross‐stage simultaneous variable selection, enabling the identification of variables that are unimportant across all stages by imposing the proposed group Lasso‐type penalty. As advantages, the proposed method is more robust against model misspecification due to its machine learning‐based approach and, in theory, enjoys the oracle property. Numerically, the optimization problem can be efficiently solved using the DC algorithm [26], where in each DC iteration, the problem can be further reduced to a simple optimization problem with the objective function being a piecewise linear function. We note that benefiting from being a machine learning‐based algorithm with an efficient numerical solver, the proposed method is well‐suited for small to moderate‐scale real‐world applications.

The remaining sections are organized as follows. In Section 2, we briefly introduce the MRL in Section 2.1 and present the details of the L1‐MRL and the choice of tuning parameters in Section 2.2 and Section 2.3. We provide theoretical justification and show that L1‐MRL yields a consistent estimator and enjoys the oracle property in Section 3. In Section 4, the performance of the method is demonstrated via extensive simulation studies and comparisons with some existing sequential methods with variable selection capability. In Section 5, we apply our method to real observational electronic health record (EHR) data from type 2 diabetes (T2D) patients. The discussion and future extensions of our work are presented in Section 6.

## 2 Method

### 2.1 Multistage Ramp Loss (MRL) for Learning Optimal DTRs

Consider a T‐stage decision‐making problem, where Y denotes the cumulative reward observed by the end of stage T, and assume that a larger value of Y indicates better treatment intervention. In precision medicine, a stage refers to a specific time point at which a treatment decision is made based on the patient's current status and history [27, 28]. Let ${\left\{A_{t}\right\}}_{t=1}^{T}$ denote the observed treatment assignment at stage t=1,…,T, with two possible treatments, denoted as {−1,+1}, available at each stage. For each t, we use $H_{t}$ to denote patients' feature variables prior to stage t and ${ℋ}_{t}\subset {\mathbb{R}}^{d_{t}}$ to denote the space of $H_{t}$. In this work, we further assume that $H_{t-1}\subset H_{t}$ for all t. A function 𝒟 is called a dynamic treatment regimen if it is a function from the functional space

$${𝒟}_{1}\times \cdots \times {𝒟}_{T}\to {\left\{-1,+1\right\}}^{T},\;{𝒟}_{t}:{ℋ}_{t}\to \{-1,+1\}.$$

The goal of optimal DTRs is to learn the optimal rules ${𝒟}^{*}=({𝒟}_{1}^{*},\ldots ,{𝒟}_{T}^{*})$ that maximize

$${𝒟}^{*}=\underset{𝒟}{argmax}\;E^{𝒟}[Y],$$

where $E^{𝒟}[\cdot ]$ denotes the expectation with $A_{t}$ forced to be ${𝒟}_{t}(H_{t})$ for t=1,…,T.

Additional causal assumptions are needed so that $E^{𝒟}[Y]$ can be estimated from observed data. To state the conditions, we use ${\bar{a}}_{t}=(a_{1},\ldots ,a_{t})\in {\left\{-1,+1\right\}}^{t}$ to denote any arbitrary fixed treatment sequence up to stage t, and let ${Ā}_{t}$ denote the observed treatment sequence correspondingly. We borrow the notation from Hernán et al. [29] and use $X({ā}_{t})$ to denote the potential outcome of the random variable X under ${Ā}_{t}={ā}_{t}$. Throughout this work, we assume that the following three assumptions hold:

Assumption 1 Stable Unit Treatment Value Assumption (SUTVA) [30]: A subject's outcome is not influenced by other subjects' treatment allocation.

Assumption 2 No Unmeasured Confounders (NUC) [31]: For any t∈{1,…,T} and ${ā}_{T}\in {\left\{-1,+1\right\}}^{T}$, we have $A_{t}⫫(H_{t+1}({\bar{a}}_{t}),\ldots ,H_{T}({\bar{a}}_{T-1}),Y({\bar{a}}_{T}))|H_{t}$.

Assumption 3 Positivity: Let ${\left\{p(A_{t}|H_{t})\right\}}_{t=1}^{T}$ denote the treatment assignment probability at each stage. There exist positive constants $0<c_{1}\leq c_{2}<1$ such that for any t=1,…,T, $c_{1}\leq p(A_{t}=1|H_{t})\leq c_{2}$ for $H_{t}$ almost surely.

Assumptions 1 to 3 are standard assumptions in many precision medicine literatures, and the implications of these conditions can be found in Chakraborty et al. [1]. In particular, Assumptions 2 and 3 will hold when the data are collected from a simple or sequential multiple assignment randomized trial (SMART) [32]. Under Assumptions 1 to 3, Qian et al. [4] show that the expected reward under an arbitrary rule 𝒟 satisfies $E^{𝒟}[Y]=E[Y\frac{\prod_{t=1}^{T}𝕀(A_{t}={𝒟}_{t}(H_{t}))}{\prod_{t=1}^{T}p(A_{t}|H_{t})}]$. Furthermore, suppose that ${𝒟}^{*}$ can be expressed by the signs of a series of optimal decision functions ${𝒟}_{t}^{*}(H_{t})=\text{sign}(f_{t}^{*}(H_{t}))$, where $f_{t}^{*}\in {ℱ}_{t}$ and ${ℱ}_{t}$ denotes the set of all measurable functions from ${ℋ}_{t}$ to ℝ. Then, learning the optimal DTRs is equivalent to solving

(1) $$(f_{1}^{*},\ldots ,f_{T}^{*})=\underset{(f_{1},\ldots ,f_{T})\in {ℱ}_{1}\times \cdots \times {ℱ}_{T}}{argmax}\;E\left[Y\frac{\prod_{t=1}^{T}𝕀(A_{t}f_{t}(H_{t})>0)}{\prod_{t=1}^{T}p(A_{t}|H_{t})}\right]$$

Note that solving (1) directly is NP‐hard and intractable in practice due to the existence of the product zero‐one loss function. To mitigate the numerical challenge, a multistage ramp loss (MRL) framework was proposed by Liu et al. [25] to substitute the product indicator function in (1). Specifically, the MRL problem aims to solve

(2) $$\begin{array}{cc} & \max_{(f_{1},\ldots ,f_{T})\in {ℱ}_{1}\times \cdots \times {ℱ}_{T}}\;\left(E\left[Y^{+}\frac{\min(\psi (A_{1}f_{1}(H_{1})),\ldots ,\psi (A_{T}f_{T}(H_{T})))}{\prod_{t=1}^{T}p(A_{t}|H_{t})}\right]\right.\\ & \left.\;+\;E\left[\sum_{\overset{(a_{1},\ldots ,a_{T})\in {\left\{-1,+1\right\}}^{T}}{\exists t\;such\;that\;a_{t}\neq A_{t}}}Y^{-}\frac{\min(\psi (a_{1}f_{1}(H_{1})),\ldots ,\psi (a_{T}f_{T}(H_{T})))}{\prod_{t=1}^{T}p(A_{t}|H_{t})}\right]\right) \end{array}$$

where $Y^{+}$ and $Y^{-}$ denote the positive and negative parts of Y, defined as $Y^{+}=\max(Y,0)$ and $Y^{-}=\max(-Y,0)$, and ψ(x)=max(min(x,1),0). The basic idea of MRL is to replace the product indicator function with the surrogate function displayed in (2). When Y is nonnegative, the surrogate function reduces to $\min(\psi (x_{1}),\ldots ,\psi (x_{T}))$, which is a smooth continuous approximation of the product indicator function and (the minimization equivalence) can be viewed as a multistage extension of the well‐known shifted ramp loss function proposed by Huang et al. [33], the remaining term is a correction term that allows Y to be negative (see section S.3.2 of the supplemental materials of Liu et al. [25] for technical details).

Lemma 2 in Liu et al. [25] shows that the surrogate problem (2) is guaranteed to yield the same optimal DTRs as the original problem under zero‐one loss. Furthermore, one major advantage of MRL over existing standard sequential DTR methods, such as Q‐learning and O‐learning, is that, unlike these methods, which must decompose the estimation of decision functions into a series of separate sub‐problems (each with respect to one stage), the MRL unifies the estimation of decision functions across all stages into a single optimization problem. In other words, the MRL can learn the optimal decision functions simultaneously across all stages. Consequently, when MRL is implemented, the update of any stage will provide feedback and update the optimal decision rules of all other stages until reaching the optimum, which can avoid the inherent accumulative error issue suffered by Q‐learning or O‐learning and yield better estimation results. This simultaneous solution is also the key advantage for identifying features that may not be important for treatment decisions across all stages, which will be elaborated on in the next section.

### 2.2 Variable Selection via Group Lasso‐Penalized MRL

To present the proposed method, from now on, we use $H_{t}$ to denote all feature variables prior to treatment at stage t, including treatment decisions from all previous stages, and assume that $H_{t}$ has been standardized for all t. We assume that the optimal decision rules are sparse in the sense that only a subset of features $H_{t}$ is important for at least one stage under the optimal rules. For convenience, we define $H_{t}=(O_{t},W_{t})$, where $W_{t}$ consists of already‐known important variables, such as treatment assignments from previous stages, which will be excluded from variable selection, and $O_{t}$ consists of a fixed length P of features considered for variable selection, which may or may not evolve over stages In practice, $O_{t}$ usually includes baseline characteristics of an individual, such as demographics and genetic information, as well as time‐varying variables, such as clinical or laboratory measurements taken at each stage t. Finally, for the purpose of feature selection, we consider that the optimal DTRs are determined by some linear functions of $H_{t}$. That is, we assume that there exists $\theta_{t}^{*}=(\alpha_{t}^{*},\beta_{t}^{*},\gamma_{t}^{*})\in {\mathbb{R}}^{d_{t}+1}$ such that the associated rules $g_{t}^{*}(H_{t})=W_{t}^{T}\alpha_{t}^{*}+O_{t}^{T}\beta_{t}^{*}+\gamma_{t}^{*}$ optimize (2). The sparsity assumption implies that only a subset of ${\left\{\beta_{t}^{*}\right\}}_{t=1}^{T}$ is non‐zero for at least one stage.

To achieve variable selection while learning the optimal rules, we consider incorporating an additional group Lasso‐type penalty into the MRL (2) to enforce sparsity over ${\left\{\beta_{t}\right\}}_{t=1}^{T}$. Specifically, we consider the following L1‐MRL optimization problem:

(3) $$\begin{array}{cc} & \max_{\theta }\;\left(E\left[Y^{+}\frac{\min(\psi (A_{1}(H_{1}^{T}\theta_{1})⁄\eta_{n}),\ldots ,\psi (A_{T}(H_{T}^{T}\theta_{T})⁄\eta_{n}))}{\prod_{t=1}^{T}p(A_{t}|H_{t})}\right]\right.\\ & \;+E\left[\sum_{\overset{(a_{1},\ldots ,a_{T})\in {\left\{-1,+1\right\}}^{T}}{\exists t\;such\;that\;a_{t}\neq A_{t}}}Y^{-}\frac{\min(\psi (a_{1}(H_{1}^{T}\theta_{1})⁄\eta_{n}),\ldots ,\psi (a_{T}(H_{T}^{T}\theta_{T})⁄\eta_{n}))}{\prod_{t=1}^{T}p(A_{t}|H_{t})}\right]\\ & \;\left.-\lambda_{n}\sum_{p=1}^{P}\sum_{t=1}^{T}\frac{\left|\beta_{tp}\right|}{\sqrt{\sum_{s=1}^{T}{\left|{\tilde{\beta }}_{sp}\right|}^{2}}}\right) \end{array}$$

Here, for succinctness, we assume that $H_{t}$ includes the intercept to simplify the notation. We also use $\left\{{\tilde{\beta }}_{tp}\right\}$ to denote $\left\{\beta_{tp}^{*}\right\}$ normalized so that $\|({\tilde{\beta }}_{t1},\ldots ,{\tilde{\beta }}_{tP}){\|}_{2}=1$ for each t. The $L_{1}$‐penalty is a typical penalty to enforce sparsity over the estimated coefficients, and the additional group Lasso‐type adaptive coefficient $1/\sqrt{\sum_{t=1}^{T}|{\tilde{\beta }}_{tp}{|}^{2}}$ aims to impose a stronger penalty on coefficients that are not significant across all stages. Like standard Lasso, $\lambda_{n}$ is the tuning parameter to control the sparsity, and we also introduce the shifting parameter $\eta_{n}\in (0,1]$ as another tuning parameter to adjust for possible model misspecification due to the linearity assumption when implementing the method. We note that when T=1, the L1‐MRL reduces to the ramp loss‐based framework studied in Huang et al. [24]. As a key observation, like the standard MRL, L1‐MRL achieves both reward maximization and variable selection in solving one unified optimization problem. Hence, L1‐MRL will still inherit the simultaneous estimation property of MRL and can attain better performance than sequential methods by avoiding cumulative false discovery and estimation error over time.

Given finite samples, we estimate $\theta^{*}=(\theta_{1}^{*},\ldots ,\theta_{T}^{*})$ by solving the empirical problem

(4) $$\begin{array}{cc} \hat{\theta } & =\underset{\theta }{argmax}\;\left(\frac{1}{n}\sum_{i=1}^{n}Y_{i}^{+}\frac{\min(\psi (A_{i1}(H_{i1}^{T}\theta_{1})⁄\eta_{n}),\ldots ,\psi (A_{iT}(H_{iT}^{T}\theta_{T})⁄\eta_{n}))}{\prod_{t=1}^{T}p(A_{it}|H_{it})}\right.\\ & \;+\frac{1}{n}\sum_{i=1}^{n}\sum_{\overset{(a_{1},\ldots ,a_{T})\in {\left\{-1,+1\right\}}^{T}}{\exists t\;such\;that\;a_{t}\neq A_{it}}}Y_{i}^{-}\frac{\min(\psi (a_{1}(H_{i1}^{T}\theta_{1})⁄\eta_{n}),\ldots ,\psi (a_{T}(H_{iT}^{T}\theta_{T})⁄\eta_{n}))}{\prod_{t=1}^{T}p(A_{it}|H_{it})}\\ & \;\left.-\lambda_{n}\sum_{p=1}^{P}\sum_{t=1}^{T}\frac{\left|\beta_{tp}\right|}{\sqrt{\sum_{s=1}^{T}{\left|{\hat{\tilde{\beta }}}_{sp}\right|}^{2}}}\right) \end{array}$$

where ${\hat{\tilde{\beta }}}_{tp}$ is an arbitrary consistent estimator of ${\tilde{\beta }}_{tp}$. As another advantage of L1‐MRL, although it is a nonconvex optimization problem, the objective function of the minimization equivalence of (4) can be decomposed as the difference between two convex functions. Hence, (4) can be solved efficiently using the DC algorithm. When the optimal decision functions are linear, as assumed in this work, in each DC iteration, the problem can be reduced to a simpler optimization problem with an objective function being a piecewise linear function. The subproblem can then be further explicitly solved by calculating the gradients of the objective function at each endpoint and performing a grid search. In particular, note that (1) is invariant if Y is subtracted by any function of $H_{1}$, so one can further refine (4) as

(5) $$\begin{array}{cc} \hat{\theta } & =\underset{\theta }{argmax}\;\left(\frac{1}{n}\sum_{i=1}^{n}{\hat{Y}}_{i}^{+}\frac{\min(\psi (A_{i1}(H_{i1}^{T}\theta_{1})⁄\eta_{n}),\ldots ,\psi (A_{iT}(H_{iT}^{T}\theta_{T})⁄\eta_{n}))}{\prod_{t=1}^{T}p(A_{it}|H_{it})}\right.\\ & \;+\frac{1}{n}\sum_{i=1}^{n}\sum_{\overset{(a_{1},\ldots ,a_{T})\in {\left\{-1,+1\right\}}^{T}}{\exists t\;such\;that\;a_{t}\neq A_{it}}}{\hat{Y}}_{i}^{-}\frac{\min(\psi (a_{1}(H_{i1}^{T}\theta_{1})⁄\eta_{n}),\ldots ,\psi (a_{T}(H_{iT}^{T}\theta_{T})⁄\eta_{n}))}{\prod_{t=1}^{T}p(A_{it}|H_{it})}\\ & \;\left.-\;\lambda_{n}\sum_{p=1}^{P}\sum_{t=1}^{T}\frac{\left|\beta_{tp}\right|}{\sqrt{\sum_{s=1}^{T}{\left|{\hat{\tilde{\beta }}}_{sp}\right|}^{2}}}\right) \end{array}$$

where we replace $Y_{i}$ by the estimated residual ${\hat{Y}}_{i}=Y_{i}-\hat{E}[Y_{i}|H_{i1}]$. This technique was first adopted in the augmented outcome‐weighted learning (AOWL) by Liu et al. [13] to improve the standard O‐learning and has shown better performance when adopted in L1‐MRL. In practice, we propose to estimate $E[Y|H_{1}]$ via standard linear or Lasso regression. A coordinate descent DC algorithm‐based procedure for solving (5) is provided in Appendix A.

Remark 1 Implementing L1‐MRL requires that treatment assignment probabilities are known in advance. This can be satisfied when the data are collected from a SMART. When ${\left\{p(A_{t}|H_{t})\right\}}_{t=1}^{T}$ are unknown, such as in an observational study, L1‐MRL can still be accommodated by substituting ${\left\{p(A_{t}|H_{t})\right\}}_{t=1}^{T}$ with consistent estimators ${\left\{\hat{p}(A_{t}|H_{t})\right\}}_{t=1}^{T}$ in (4) or (5), assuming that ${\left\{p(A_{t}|H_{t})\right\}}_{t=1}^{T}$ can be consistently estimated from the observed data.

### 2.3 Select Optimal Tuning Parameters

One needs to prespecify the choice of tuning parameters $\left(\lambda_{n},\eta_{n}\right)$ when implementing the L1‐MRL. In this work, we propose to select the optimal tuning parameters through cross‐validation and consider the following AIC‐type criterion [34]:

(6) $$n*\log(\frac{\hat{ℛ}(\hat{\theta }(\lambda_{n},\eta_{n}),\eta_{n})}{\hat{ℛ}(\hat{\theta }(0,\eta_{n}),\eta_{n})})-k(\lambda_{n},\eta_{n})$$

Here,

$$\begin{array}{cc} \hat{ℛ}(\theta ,\eta ) & =\sum_{i=1}^{n}Y_{i}^{+}\frac{\min(\psi (A_{i1}(H_{i1}^{T}\theta_{1})⁄\eta ),\ldots ,\psi (A_{iT}(H_{iT}^{T}\theta_{T})⁄\eta ))}{\prod_{t=1}^{T}p(A_{it}|H_{it})}\\ & \;+\sum_{i=1}^{n}\sum_{\overset{(a_{1},\ldots ,a_{T})\in {\left\{-1,+1\right\}}^{T}}{\exists t\;such\;that\;a_{t}\neq A_{it}}}Y_{i}^{-}\frac{\min(\psi (a_{1}(H_{i1}^{T}\theta_{1})⁄\eta ),\ldots ,\psi (a_{T}(H_{iT}^{T}\theta_{T})⁄\eta ))}{\prod_{t=1}^{T}p(A_{it}|H_{it})} \end{array}$$

denotes the empirical estimator of the MRL objective function with coefficients θ and shifting parameter η. $\hat{\theta }(\lambda ,\eta )$ denotes the estimated coefficients of (4) or (5) under the tuning pair (λ,η), and k(λ,η) denotes the number of non‐zero coefficients of $\hat{\theta }(\lambda ,\eta )$. Similar BIC‐type criteria were adopted in Shi et al. [17] under an A‐learning framework and in He et al. [23] under a doubly robust O‐learning framework. When implementing L1‐MRL, we select the optimal tuning parameters that maximize (6) on testing data under cross‐validation from the prespecified tuning grid.

## 3 Theoretical Result

We show that the estimated rules learned via L1‐MRL are consistent and enjoy the oracle property under mild conditions. In this section, we fix the shifting parameter η=1 and let the first P entries of $\theta_{t}$ correspond to the coefficients of $O_{t}$, which are sparse and considered for variable selection. We establish the result for a more general penalty term:

$$\rho_{\lambda_{n},\gamma }(\theta )=\lambda_{n}\sum_{\begin{array}{l} 1\leq t\leq T\\ 1\leq p\leq P \end{array}}\omega_{p,\gamma }\left|\beta_{t,p}\right|,$$

where γ is a proper fixed positive constant and $\omega_{p,\gamma }={\left(\sum_{t=1}^{T}{\left|{\hat{\tilde{\beta }}}_{t,p}\right|}^{2}\right)}^{-\frac{\gamma }{2}}$. The penalty term in L1‐MRL is a special case of $\rho_{\lambda_{n},\gamma }(\theta )$ when γ=1.

To state the additional necessary condition, we define:

(7) $$\begin{array}{cc} ℒ(\theta ) & =-\left(E\left[Y^{+}\frac{\min(\psi (A_{1}H_{1}^{T}\theta_{1}),\ldots ,\psi (A_{T}H_{T}^{T}\theta_{T}))}{\prod_{t=1}^{T}p(A_{t}|H_{t})}\right]\right.\\ & \left.\;+\;E\left[\sum_{\overset{(a_{1},\ldots ,a_{T})\in {\left\{-1,+1\right\}}^{T}}{\exists t\;such\;that\;a_{t}\neq A_{t}}}Y^{-}\frac{\min(\psi (a_{1}H_{1}^{T}\theta_{1}),\ldots ,\psi (a_{T}H_{T}^{T}\theta_{T}))}{\prod_{t=1}^{T}p(A_{t}|H_{t})}\right]\right) \end{array}$$

so $\theta^{*}$ is the minimizer of ℒ(θ) by definition. In addition to Assumptions 1 to 3, we assume that the following assumption holds:

Assumption 4 ℒ(θ) is three times differentiable and has a unique minimizer $\theta^{*}$. Moreover, $\nabla^{2}ℒ(\theta^{*})≻0$ and the third‐order derivatives of ℒ(θ) are bounded.

Assumption 4 is a regularity assumption similar to the conditions introduced in Fan et al. [35] for establishing the consistency and the oracle property of the SCAD penalty. Essentially, Assumption 4 ensures that the objective function is locally strictly convex near the optimal solution $\theta^{*}$. Under this additional assumption, we obtain the following theorem:

Theorem 1 *Assume that* ${\left\{ℋ\right\}}_{t=1}^{T}$ *are compact and Assumptions* *1* *to* *4* *hold. Let* $\left\{{\hat{\tilde{\beta }}}_{t,p}\right\}$ *be* $\sqrt{n}-$ *consistent estimators of* $\left\{{\tilde{\beta }}_{t,p}\right\}$ *, and* $\lambda_{n}n^{\frac{1}{4}-\frac{\xi }{2}}=o(1)$ *for some* $\xi \in (0,\frac{1}{2})$ *. Then, there exists a local minimizer* $\hat{\theta }$ *of* (*4*) *such that* $\left\|\theta^{*}-\hat{\theta }{\|}_{2}=O_{p}(n^{-\frac{1}{4}+\frac{\xi }{2}}\right)$ *. Moreover, suppose that* $\lambda_{n}n^{\frac{1}{4}-\frac{\xi^{\prime }}{2}+\frac{\gamma }{2}}\to \infty$ *also holds for some* $\xi <\xi^{\prime }$ *, then we have*

$$\begin{array}{ll} & \lim_{n\to \infty }P\left(\max\{\left|{\hat{\beta }}_{t,p}\right|:\beta_{t,p}^{*}=0\;\text{for}\text{some}\text{fixed}p\text{and}\text{all}t=1,\ldots ,T\}=0\right)=1. \end{array}$$

The first part of Theorem 1 indicates that as n goes to infinity, there always exists a local minimizer of (4) within the ball of radius $O_{p}(n^{-\frac{1}{4}+\frac{\xi }{2}})$ centered at the true optimal solution $\theta^{*}$. Furthermore, when the additional requirement on $\lambda_{n}$ is also satisfied, the second part of the theorem shows that $\hat{\theta }$ enjoys the oracle property in the sense that variables that are insignificant across all stages under the optimal rules will have estimated coefficients strictly equal to 0 with probability 1 as n goes to infinity. Therefore, L1‐MRL is guaranteed to eliminate variables that are insignificant across all stages and identify the union of variables that are important for at least one stage. The proof of Theorem 1 is presented in Appendix B.

Remark 2 Theorem 1 establishes the oracle property for L1‐MRL simultaneously across all stages. This differs from existing sequential approaches, where the oracle property is also established in a stagewise manner. For oracle property results of some existing sequential DTR methods with variable selection capability, we refer to Song et al. [21], Shi et al. [17], and Bian et al. [18].

Remark 3 Theorem 1 requires that one can obtain $\sqrt{n}$‐consistent estimators of $\left\{{\tilde{\beta }}_{t,p}\right\}$ to construct the adaptive coefficients in (4). When consistent estimators are difficult to obtain, alternatively, the proposed group Lasso‐type penalty can be replaced by other penalty terms that also enjoy the oracle property. Examples include the MCP [36] or SCAD penalty [35].

## 4 Simulation Studies

We conduct simulation studies to assess the performance of L1‐MRL in this section. We consider a two‐stage SMART design and first generate 12 time‐independent baseline covariates $\left(Z_{1},\ldots ,Z_{12}\right)$ from a multivariate normal distribution with mean 0, variance 1, and covariance structure:

$$Cov(Z_{i},Z_{j})=\left\{\begin{array}{ll} 0.2,\; & \forall 1\leq i<j\leq 6,\\ 0,\; & \text{for all other}\;i\neq j. \end{array}\right.$$

In addition, we generate three time‐dependent covariates, denoted as $\left(X_{11},X_{12}\right)$, $\left(X_{21},X_{22}\right)$ and $\left(X_{31},X_{32}\right)$, independently according to

$$\begin{array}{l} X_{i1}=Z_{1}\omega_{i}+\alpha_{i}+\epsilon_{i1},\;X_{i2}=Z_{1}\omega_{i}+\alpha_{i}(1+A_{1}/2)+\epsilon_{i2}, \end{array}$$

where ${\left\{\omega_{i}\right\}}_{i=1,2,3}$ are independently sampled from a uniform distribution Unif[0,1], ${\left\{\alpha_{i}\right\}}_{i=1,2,3}$ are independently sampled from a standard normal distribution N(0,1), and ${\left\{(\epsilon_{i1},\epsilon_{i2})\right\}}_{i=1,2,3}$ are independent noise terms sampled from a standard normal distribution N(0,1).

We consider the following two settings:

- **Setting 1:**
$$\begin{array}{ll} Y & =1+Z_{1}+Z_{3}+A_{1}(3Z_{1}+3Z_{2}-2Z_{7}-2Z_{8}-2X_{11})\\ & \;+A_{2}(3Z_{1}+3Z_{2}-2Z_{7}-2Z_{8}-2X_{12})+\epsilon_{Y}. \end{array}$$
- **Setting 2:**
$$\begin{array}{ll} Y & =1+Z_{1}+Z_{2}+\frac{1}{2}(Z_{3}^{2}+Z_{4}^{2})\\ & \;+A_{1}\left({\left(Z_{1}+3\right)}^{2}+{\left(Z_{2}+3\right)}^{2}+{\left(Z_{7}-3\right)}^{2}\right.\\ & \;\left.+{\left(Z_{8}-3\right)}^{2}+\frac{2}{3}{\left(X_{11}-5\right)}^{2}-60\right)\\ & \;+A_{2}\left({\left(Z_{1}+3\right)}^{2}+{\left(Z_{2}+3\right)}^{2}+{\left(Z_{7}-3\right)}^{2}\right.\\ & \;\left.+{\left(Z_{8}-3\right)}^{2}+\frac{2}{3}{\left(X_{12}-5\right)}^{2}-60\right)+\epsilon_{Y}. \end{array}$$

For both settings, $\epsilon_{Y}$ is generated from a standard normal distribution N(0,1), and treatments are assigned following the regression model:

$$\begin{array}{ll} & \text{logit}\;P(A_{1}=1|Z,X_{11},X_{21},X_{31})=\frac{1}{3}X_{11},\;\\ & \text{logit}\;P(A_{2}=1|Z,X_{12},X_{22},X_{32},A_{1})=\frac{1}{3}X_{12}+\frac{1}{2}A_{1} \end{array}$$

When conducting the simulation, we assume that the treatment assignment models are unknown and need to be estimated. The feature variables for each stage are set to be:

$$\begin{array}{ll} & H_{1}=(Z_{1},\ldots ,Z_{12},X_{11},X_{21},X_{31}),\;\;\\ & H_{2}=(Z_{1},\ldots ,Z_{12},X_{12},X_{22},X_{32},A_{1}), \end{array}$$

and are standardized during the estimation. We repeat the analyses 500 times for both settings with training sample sizes of N=200 and 400, respectively.

For L1‐MRL, we solve the refined problem (5) and select the optimal tuning parameters from the tuning grid $(\lambda_{n},\eta_{n})\in 2^{0:10}\times 10^{-3:-5}$ via twofold cross‐validation according to (6). We use standard Lasso regression to estimate the conditional mean model $E[Y|H_{1}]$. When estimating the coefficients under tuning parameters $\left(\lambda_{n},\eta_{n}\right)$, we select the estimated coefficients of (5) under $\left(0,\eta_{n}\right)$ as the initial point of the DC iteration and use the solution of AOWL as the initial point under $\left(0,\eta_{n}\right)$. We also use the solution from AOWL to construct the adaptive coefficients in (5). For the second stage, $A_{1}$ is excluded from variable selection, leaving P=15 with only 12 time‐independent and three time‐dependent covariates considered for variable selection.

To demonstrate the performance, we also compare L1‐MRL with the following four competing sequential methods with variable selection capability:

- *Q‐learning with* $L_{1}$ *‐penalty* [4]: Assume that the Q‐function, which is defined as
$$\begin{array}{l} Q_{t}(h_{t},a_{t})=E\left[\left.\max_{a_{t+1}\in \{-1,+1\}}Q_{t+1}(H_{t+1},a_{t+1})\right|H_{t}=h_{t},A_{t}=a_{t}\right] \end{array}$$
  and $Q_{T+1}=Y$, follows linear regression models $Q_{t}(H_{t},A_{t})=H_{t}^{T}\omega_{t}+A_{t}H_{t}^{T}\theta_{t}$. Then, it can be shown that:
$${𝒟}_{t}^{*}(H_{t})=\text{sign}(Q_{t}(H_{t},1)-Q_{t}(H_{t},-1)).$$
  almost surely. Q‐learning with $L_{1}$‐penalty [4] incorporates variable selection and learns the optimal decision functions by estimating $Q_{t}(H_{t},A_{t})$ via standard Lasso regression through backward induction from t=T to t=1.
- *A‐learning via Danzig selector* [17]: The A‐learning approach assumes that the Q‐function satisfies $Q_{t}(H_{t},A_{t})=H_{t}^{T}\omega_{t}+\frac{1}{2}(1+A_{t})H_{t}^{T}\theta_{t}$. In Shi et al. [17], $Q_{t}(H_{t},A_{t})$ is estimated via solving a Dantzig selector [37] stagewise in backward order, which imposes an $L_{1}$‐penalty on the estimated coefficients.
- *Dynamic weight least squares (dWOLS)* [18]: Bian et al. propose the dynamic weighted ordinary least squares approach [5] to learn optimal DTRs by modeling the Q‐function, assuming $Q_{t}(H_{t},A_{t})=H_{t}^{T}\omega_{t}+\frac{1}{2}(1+A_{t})H_{t}^{T}\theta_{t}$, similar to A‐learning. The method incorporates variable selection by imposing a hierarchical Lasso‐type penalty for each stage separately in backward order.
- *O‐learning with* $L_{1}$ *‐penalty*: Zhao et al. [8] propose estimating optimal DTRs by solving a series of weighted support vector machine (SVM) problems [38]:
$$\begin{array}{l} {\hat{\theta }}_{t}=\underset{\theta }{argmin}\;\left(\sum_{i=1}^{n}\frac{\prod_{s=t+1}^{T}𝕀(A_{is}H_{is}^{T}{\hat{\theta }}_{s}>0)}{\prod_{s=t}^{T}p(A_{is}|H_{is})}\phi (A_{it}H_{it}^{T}\theta )+\lambda_{n}{\left\|\theta \right\|}_{2}^{2}\right) \end{array}$$
  via backward induction, where ϕ(·) denotes the hinge loss function defined as $\phi (x)={\left(1-x\right)}_{+}$. To incorporate variable selection, we consider the modified $L_{1}$‐penalized O‐learning and estimate the coefficients by solving:
$$\begin{array}{l} {\hat{\theta }}_{t}=\underset{\theta }{argmin}\;\left(\sum_{i=1}^{n}\frac{\prod_{s=t+1}^{T}𝕀(A_{is}H_{is}^{T}{\hat{\theta }}_{s}>0)}{\prod_{s=t}^{T}p(A_{is}|H_{is})}\phi (A_{it}H_{it}^{T}\theta )+\lambda_{n}{\left\|\theta \right\|}_{1}\right) \end{array}$$
  also in backward order, similar to Li et al. [22]. In the final simulation study, we implement AOWL, where all standard weighted SVM problems are replaced by corresponding $L_{1}$‐penalized weighted SVM problems.

In this section and in Section 5 later, A‐learning is implemented using the function *PAL()* from the R package *ITRSelect*, and Q‐learning is implemented using the function *ql()* from the R package *DTRlearn2*. The dWOLS is conducted using an R program modified from the supplementary GitHub repository of Bian et al. (https://github.com/ZeyuBian/pdwols), and O‐learning is conducted by solving the optimization problem directly using the linear programming solver *lp()* from the R package *lpSolve*. For A‐learning, Q‐learning, and dWOLS, the tuning parameters are selected via cross‐validation from the program's default tuning grid. In particular, for dWOLS, the hyperparameter α is fixed at 0.5, analogous to the choice in Bian et al. (see sect. 2 of Bian et al. for more details on the tuning parameter setup). For O‐learning, the tuning parameter $\lambda_{n}$ is selected from the prespecified tuning grid $10^{-5:5}$ via twofold cross‐validation. For L1‐MRL and competing methods that require the treatment assignment probability to be known in advance, we use Lasso logistic regression to estimate the treatment assignment probability using $H_{t}$ as predictors for each stage.

For each method, we evaluate the performance of treatment optimization by comparing the expected reward under the estimated rules on an independent testing dataset of size 5000 using the Monte Carlo method based on the true models. In addition, we report the number of false negatives (FN) and false positives (FP) among $O_{t}$, as well as the overall false discovery rate (FDR), defined as (FN+FP)/P, that is, the proportion of feature variables that are falsely selected or removed, at each stage to assess the variable selection performance of each method. As an ideal method is expected to yield stable variable selection when repeatedly implemented in practice, we also report the average pairwise Jaccard index (JI) between the sets of selected variables under 500 repeated analyses. The Jaccard index between two repeated analyses is defined as:

$$J(S_{1},S_{2})=\frac{\left|S_{1}\cap S_{2}\right|}{\left|S_{1}\cup S_{2}\right|},$$

and $S_{1}$ and $S_{2}$ denote the sets of selected important variables among P=15 candidate variables under two repeated analyses. We let $J(S_{1},S_{2})=1$ when $S_{1}=S_{2}=\emptyset$. By definition, a Jaccard index closer to 1 indicates higher similarity between selected variables and more stable variable selection. The simulation results for the two simulation settings are reported in Tables 1 and 2, respectively.

**TABLE 1:** Summary of testing reward, the number of false negatives (FN), false positives (FP), false discovery rate (FDR), and the Jaccard index (JI) for simulation setting 1.

N	Method	Testing reward	Stage 1				Stage 2			
			FN^a^	FP^a^	FDR	JI	FN	FP	FDR	JI
200	L1‐MRL	8.329 (0.983)	0.766 (0.902)	0.754 (1.301)	0.101 (0.092)	0.615	0.676 (0.947)	1.488 (1.070)	0.144 (0.082)	0.679
	A‐learning	9.042 (0.491)	0.700 (0.641)	0.100 (0.344)	0.053 (0.049)	0.746	1.026 (0.723)	0.128 (0.379)	0.077 (0.057)	0.669
	Q‐learning	5.493 (1.239)	4.094 (1.193)	1.416 (1.946)	0.367 (0.099)	0.177	0.000 (0.000)	4.198 (2.197)	0.280 (0.146)	0.586
	O‐learning	6.690 (0.364)	3.062 (0.301)	3.956 (1.781)	0.468 (0.119)	0.414	3.010 (0.100)	3.920 (1.990)	0.462 (0.133)	0.427
	dWOLS	5.508 (1.529)	0.408 (1.060)	8.802 (2.417)	0.614 (0.113)	0.802	0.632 (1.293)	7.264 (4.322)	0.526 (0.240)	0.577
400	L1‐MRL	9.364 (0.448)	0.120 (0.397)	0.602 (1.093)	0.048 (0.074)	0.811	0.148 (0.418)	1.336 (0.825)	0.099 (0.058)	0.858
	A‐learning	9.514 (0.369)	0.282 (0.489)	0.004 (0.063)	0.019 (0.033)	0.896	0.594 (0.640)	0.022 (0.147)	0.041 (0.044)	0.799
	Q‐learning	5.289 (1.297)	3.834 (1.339)	1.624 (1.838)	0.364 (0.107)	0.140	0.000 (0.000)	4.298 (2.087)	0.287 (0.139)	0.585
	O‐learning	6.962 (0.157)	3.026 (0.159)	3.398 (2.227)	0.428 (0.146)	0.422	3.004 (0.063)	3.422 (2.211)	0.428 (0.147)	0.428
	dWOLS	6.046 (1.335)	0.034 (0.391)	9.892 (0.866)	0.662 (0.035)	0.981	0.136 (0.625)	9.800 (2.710)	0.662 (0.157)	0.848

*Note:* Testing reward, FN, FP, and FDR are reported in the *mean (standard deviation)* format.

^a^ A feature variable is identified as significant if the absolute value of its corresponding coefficient in the decision rule is greater than $10^{-6}$. FN counts the number of insignificant variables among $\left(Z_{1},Z_{2},Z_{7},Z_{8},X_{1}\right)$, and FP counts the number of significant variables among (Z,X) other than $\left(Z_{1},Z_{2},Z_{7},Z_{8},X_{1}\right)$.

**TABLE 2:** Summary of testing reward, the number of false negatives (FN), false positives (FP), false discovery rate (FDR), and the Jaccard index (JI) for simulation setting 2.

N	Method	Testing reward	Stage 1				Stage 2			
			FN^a^	FP^b^	FDR	JI	FN	FP	FDR	JI
200	L1‐MRL	21.782 (2.322)	0.762 (0.914)	0.938 (1.436)	0.113 (0.100)	0.585	0.574 (0.866)	1.594 (1.145)	0.145 (0.083)	0.676
	A‐learning	19.676 (2.010)	2.098 (0.796)	0.040 (0.225)	0.143 (0.056)	0.446	2.404 (0.833)	0.022 (0.147)	0.162 (0.057)	0.470
	Q‐learning	13.088 (2.518)	4.284 (1.127)	1.162 (1.752)	0.363 (0.087)	0.253	0.000 (0.000)	3.960 (2.011)	0.264 (0.134)	0.587
	O‐learning	11.794 (1.182)	3.318 (0.479)	3.572 (2.128)	0.459 (0.140)	0.352	3.246 (0.471)	3.462 (2.573)	0.447 (0.162)	0.361
	dWOLS	11.018 (2.991)	1.226 (1.401)	6.136 (3.253)	0.491 (0.165)	0.476	1.742 (1.822)	4.900 (3.706)	0.443 (0.171)	0.326
400	L1‐MRL	24.175 (1.353)	0.164 (0.440)	0.592 (1.035)	0.050 (0.071)	0.796	0.102 (0.363)	1.378 (0.928)	0.099 (0.064)	0.866
	A‐learning	21.148 (1.965)	1.626 (0.841)	0.006 (0.077)	0.109 (0.056)	0.564	2.116 (0.810)	0.004 (0.063)	0.141 (0.054)	0.561
	Q‐learning	12.508 (2.867)	3.878 (1.369)	1.646 (1.979)	0.368 (0.098)	0.174	0.000 (0.000)	4.144 (2.119)	0.276 (0.141)	0.585
	O‐learning	12.635 (0.748)	3.202 (0.412)	3.380 (2.027)	0.439 (0.135)	0.372	3.094 (0.312)	3.062 (2.322)	0.410 (0.153)	0.404
	dWOLS	11.217 (3.083)	1.302 (1.736)	6.116 (4.161)	0.495 (0.187)	0.46	1.654 (2.003)	5.044 (4.216)	0.447 (0.193)	0.348

*Note:* Testing reward, FN, FP, and FDR are reported in *mean (standard deviation)* format.

^a^ A feature variable is identified as significant if the absolute value of its corresponding coefficient in the decision rule is greater than $10^{-6}$. FN counts the number of insignificant variables among $\left(Z_{1},Z_{2},Z_{7},Z_{8},X_{1}\right)$, and FP counts the number of significant variables among (Z,X) other than $\left(Z_{1},Z_{2},Z_{7},Z_{8},X_{1}\right)$.

From Table 1, we first note that L1‐MRL and A‐learning tend to produce higher estimated testing rewards, and lower FN and FP, resulting in a lower overall FDR, compared to the other three competing methods under both N=200 and N=400. This demonstrates that L1‐MRL and A‐learning can achieve superior performance in both treatment optimization and variable selection. In terms of stability, with the exception of dWOLS, which yields a much higher JI during Stage 1 at the cost of significantly higher FP, L1‐MRL, and A‐learning also yield overall higher JI, indicating more stable variable selection performance. When comparing A‐learning with L1‐MRL, we note that the performance of L1‐MRL is relatively worse, with slightly lower rewards and a higher number of false discoveries, particularly when the sample size is small. However, it is worth noting that the regression models for regression‐based approaches, including A‐learning, are nearly correctly specified in this setting, with treatment assignment models also correctly specified by design. This suggests that L1‐MRL, as a machine learning‐based approach, is capable of achieving better or comparable performance to regression‐based approaches when the models are nearly correctly specified. Regarding the performance of L1‐MRL, the lower FDR demonstrates that L1‐MRL exhibits the oracle property. Moreover, L1‐MRL tends to produce a lower FDR in the first stage than in the second stage, whereas sequential methods, such as Q‐learning, O‐learning, and dWOLS, tend to yield a higher FDR in the first stage than in the second stage. This provides numerical evidence suggesting that sequential methods are more prone to accumulative errors, whereas L1‐MRL is better able to avoid accumulative errors and false discoveries compared to sequential methods, benefiting from its simultaneous estimation property.

For the second simulation setting, Table 2 again shows that L1‐MRL and A‐learning achieve superior performance compared to the other three competing methods, both in reward optimization and variable selection, under both sample sizes. However, L1‐MRL now outperforms A‐learning, with higher testing rewards and lower FDR. By design, $Z_{1}$ is an important variable in the second simulation setting, as it correlates with both $X_{1}$ and $Z_{2}$ and, consequently, directly and indirectly influences the cumulative reward. When checking the selection rate of $Z_{1}$ under N=400, A‐learning is less inclined to select $Z_{1}$ as an important variable, with only a 35.8% and 11.2% selection rate for the first and second stages, respectively, whereas L1‐MRL is more likely to select $Z_{1}$, with selection rates of more than 90% in both stages. Note that Y is now generated according to a nonlinear function of the feature variables, so the decision rule models are misspecified. This suggests that A‐learning's performance will be negatively impacted by model misspecification, resulting in over‐conservative, suboptimal rules. In comparison, L1‐MRL is designed to maximize (a surrogate function of) the expected reward directly, making it more robust and better able to maintain high rewards despite model misspecification. Regarding L1‐MRL, the lower FDR indicates that L1‐MRL can also maintain the oracle property under misspecified models. Moreover, L1‐MRL continues to produce a lower FDR in the first stage than in the second stage, whereas regression approaches, such as Q‐learning, O‐learning, and dWOLS, continue to produce higher FDR in the first stage than in the second stage. This suggests that L1‐MRL still benefits from its simultaneous estimation property and can avoid cumulative errors under misspecified models, in contrast to sequential approaches.

To summarize, the two simulation studies suggest that L1‐MRL and A‐learning tend to outperform the other three competing methods in terms of both reward optimization and variable selection. When the decision functions are nearly correct, L1‐MRL tends to achieve comparable, though slightly worse, performance than A‐learning, particularly when the sample size N is small. However, when models are misspecified, L1‐MRL is more robust against model misspecification and can achieve superior performance compared to A‐learning and other competing methods. In terms of computational cost, the average time to run one twofold cross‐validation for L1‐MRL with N=200 and N=400 is 0.460 and 0.801 min under setting 1, and 0.444 and 0.731 min under setting 2, respectively. Consequently, L1‐MRL is more computationally expensive than regression‐based approaches and is better suited for applications with a moderate sample size.

## 5 Application to T2D EHR Data

We apply L1‐MRL to an observational electronic health record (EHR) dataset of type 2 diabetes (T2D) patients. The raw dataset consists of EHR data for 55,246 T2D patients from the Ohio State University Hospital system between 2008 and 2018. Since medication regimens for T2D patients should be reevaluated every 3‐6 months in practice [39], we infer patients' treatment stages and medications received from their medication prescription records. Specifically, any two consecutive prescriptions occurring less than 90 days apart, which are more likely to represent the same treatment, are combined and counted as part of the same treatment stage (extending the treatment ending time accordingly). A visualized example is provided in the Supporting Information to further illustrate the treatment inference procedure. In the screening stage, we require that qualified candidate patients satisfy the following criteria:

1. Patients must have had two identifiable treatment stages since 2008.
2. The duration of both the first and second treatment stages must be longer than 180 days.
3. Patients' inferred treatment in the first stage must be either fast‐acting insulin monotherapy or fast‐acting insulin plus long‐acting insulin combined therapy.
4. Let $T_{1}$ and $T_{2}$ denote the initial times of the first and second‐stage treatments, respectively, and $T_{3}=T_{2}+\text{180 days}$. Patients must have at least one HbA1c laboratory test result within the time interval $\left[T_{i}-\text{90 days},T_{i}+\text{90 days}\right]$ for i=1,2,3.

As will be stated later, in this study, we aim to learn the optimal DTRs that lead to improved HbA1c reduction for the selected cohort. HbA1c level is one of the main glycemic control targets for T2D patients [40]. As HbA1c reflects the average blood glucose level over the past three months [41], the first and second criteria ensure that the treatment effect would be visible from changes in HbA1c levels. By imposing the third criterion, we restrict our attention to patients whose inferred treatment in Stage 1 is either fast‐acting insulin (F) or fast‐acting plus long‐acting insulin (FI) combined therapy. These two treatments are the two most frequently received initial treatments in the observed dataset, with evidence indicating their effectiveness in glucose control. The last criterion ensures that the HbA1c level at the time points of interest can be reliably approximated from available laboratory test results.

After screening, 381 patients satisfied the requirements. To conduct the study, we generate a two‐stage dataset with two arms available at each stage, defined as follows:

• Stage 1:
  ◦ Fast‐acting insulin (F) arm: Patients who adopted fast‐acting insulin monotherapy.
  ◦ Fast‐acting + long‐acting insulin (FL) arm: Patients who adopted fast‐acting insulin plus long‐acting insulin combined therapy.
• Stage 2:
  ◦ Intensified arm (I): For the fast‐acting insulin arm, patients are classified into the intensified arm if they continued using fast‐acting insulin plus long‐acting insulin or added at least one other type of T2D medication. For the fast‐acting + long‐acting insulin arm, patients are classified into the intensified arm if they continued using fast‐acting and long‐acting insulin plus at least one other type of T2D medication.
  ◦ Maintained/Reduced arm (MR): For the fast‐acting insulin arm, patients are classified into the maintained/reduced arm if they maintained fast‐acting insulin monotherapy or stopped using fast‐acting insulin. For the fast‐acting plus long‐acting insulin arm, patients are classified into the maintained/reduced arm if they maintained fast‐acting plus long‐acting insulin combined treatment or stopped using either fast‐acting or long‐acting insulin.

For each patient, we extract three time‐independent variables, including baseline age, gender, and baseline smoking status, plus six time‐dependent variables: BMI, systolic blood pressure, low‐density lipoprotein (LDL) level, high‐density lipoprotein (HDL) level, triglyceride level, and HbA1c level measured at the beginning of each treatment stage.

For time‐dependent variables other than HbA1c, the value at each time point is approximated via linear interpolation using the most recent laboratory test results before and after the time point to reduce the impact of drastic changes in imputed values. For patients with insufficient laboratory test records, the values are imputed using the population mean. To improve the reliability of the analysis, LDL, HDL, and triglyceride levels are truncated at 99%, 99%, and 97.5%, respectively, to eliminate the impact of rare extreme values. For HbA1c levels, we use $\left(Y_{1},Y_{2},Y_{3}\right)$ to denote the HbA1c levels at $\left(T_{1},T_{2},T_{3}\right)$, respectively. As the HbA1c level reflects the average blood glucose level over the past three months as mentioned earlier, we use the observed laboratory test result closest to $T_{i}$ within the time interval $\left[T_{i}-\text{90 days},T_{i}+\text{90 days}\right]$ to approximate $Y_{i}$ for i=1,2,3. This is guaranteed to be feasible for the selected cohort by the fourth screening criterion.

In this study, the cumulative reward Y is set to be $Y=-(\frac{Y_{3}-Y_{1}}{T_{3}-T_{1}})*365$, which represents the cumulative HbA1c reduction at 180 days after the initiation of the second‐stage treatment, rescaled to 1 year. Thus, a higher Y indicates better treatment performance. We let the first‐stage feature variables $H_{1}$ consist of the three time‐independent variables and the six time‐dependent variables measured at $T_{1}$. Similarly, $H_{2}$ consists of the three time‐independent variables and the six time‐dependent variables measured at $T_{2}$, plus the treatment assignment of the first stage and the duration of the first‐stage treatment in days.

We implement L1‐MRL and compare its performance with four competing methods explored in the simulation studies. To mitigate variability within the observed data and assess the performance of each method fairly and objectively, we repeatedly sample 50% of patients as training data and evaluate the performance using the remaining 50% as testing data over 500 iterations. The performance of each estimated rule is evaluated based on the expected HbA1c reduction on the testing data, along with the number of selected variables at each stage and the Jaccard index between repeated analyses. As the data are observational, we estimate the testing reward using the stabilized inverse probability‐weighted estimator (SIPWE) [8], where the SIPWE for T=2 and decision rules $f=(f_{1},f_{2})$ is defined as

$$\begin{array}{cc} \hat{𝒱}(f) & =\left(\sum_{i=1}^{n}Y_{i}\frac{𝕀(A_{i1}f_{1}(H_{i1})>0)𝕀(A_{i2}f_{2}(H_{i2})>0)}{p(A_{i1}|H_{i1})p(A_{i2}|H_{i2})}\right)\\ & \;/\left(\sum_{i=1}^{n}\frac{𝕀(A_{i1}f_{1}(H_{i1})>0)𝕀(A_{i2}f_{2}(H_{i2})>0)}{p(A_{i1}|H_{i1})p(A_{i2}|H_{i2})}\right) \end{array}$$

To implement SIPWE, we use Lasso logistic regression to estimate the unknown treatment assignment probability using $H_{t}$ as predictors and all samples as training data for t=1,2. To further reduce the variability of the estimated reward on testing data during repeated sampling and obtain reliable comparisons, we exclude subjects with extreme weighted rewards by truncating $Y/(p(A_{1}|H_{1})p(A_{2}|H_{2}))$ at 10. After two stages of truncation, 335 patients remained in the final analysis.

Remark 4 In real‐world applications, L1‐MRL only needs to be implemented once on all available training data.

The implementation of L1‐MRL and other competing methods follows the same description as in Section 4, except that for L1‐MRL, we fix the adaptive coefficient to be the coefficient calculated from the solution of AOWL using all data as training data. Additionally, for both the training stage and the testing reward estimation, the treatment assignment probability is estimated only once and fixed using all available samples as training data via Lasso logistic regression.

The real data application results are displayed in Table 3. From the table, we first observe that L1‐MRL yields higher rewards than both all possible one‐size‐fits‐all rules and competing methods. This demonstrates that L1‐MRL preserves the reward optimization capability through treatment individualization while considering variable selection and outperforms the competing methods in a real application scenario. In terms of variable selection, L1‐MRL tends to select more variables than Q‐learning and dWOLS. However, by examining the frequency table of each candidate variable being selected as important (the complete table is provided as Table S.2 in the Supporting Information), the decision rules learned by Q‐learning and dWOLS are less practically meaningful, with no clear preference for any candidate features in either one or both stages. Compared with A‐learning and O‐learning, L1‐MRL is able to produce higher rewards by selecting fewer important variables, with also a higher JI indicating a more stable variable selection. Moreover, by reviewing Table S.2 again, L1‐MRL shows a clear and stronger inclination to select HbA1c and BMI as important features for both stages. Apart from the definition of the cumulative reward Y, domain evidence has shown that HbA1c is widely recognized and adopted as one of the most important health biomarkers for tailoring treatments for T2D patients [42], and studies have also revealed that overweight status affects the response to insulin‐involved therapy in T2D patients [43]. The stronger inclination of L1‐MRL to select these two features implies that the variable selection results of L1‐MRL are also more practically reasonable, as they align with existing domain evidence.

**TABLE 3:** Summary of the real data application results and the expected HbA1c reduction under the four possible one‐size‐fits‐all rules.

Method	Testing HbA1c reduction (%)	Number of selected variables stage 1^a^	JI stage 1	Number of selected variables stage 2^a^	JI stage 2
L1‐MRL	0.215 (0.135)	2.685 (2.354)	0.327	2.315 (2.369)	0.350
A‐learning	0.185 (0.131)	2.573 (1.076)	0.292	3.393 (1.748)	0.296
Q‐learning	0.189 (0.118)	1.379 (2.135)	0.423	0.685 (1.110)	0.451
O‐learning	0.162 (0.111)	3.142 (2.272)	0.291	2.852 (2.515)	0.290
dWOLS	0.207 (0.111)	1.599 (1.073)	0.659	0.004 (0.090)	0.996

	FL‐I^b^	FL‐MR	F‐I	F‐MR
Testing HbA1c Reduction (%)	0.180 (0.116)^c^	0.185 (0.092)	0.039 (0.077)	0.085 (0.083)

^a^ *Number of selected variables* counts the number of variables identified as significant among the three time‐independent and six time‐dependent feature variables. A feature variable is identified as significant if the absolute value of its corresponding estimated coefficient is greater than $10^{-6}$.

^b^ FL‐I denotes the naive one‐size‐fits‐all rule, where all patients are treated with fast‐acting insulin plus long‐acting insulin during stage 1 and receive intensified treatment during Stage 2 (FL‐MR, F‐I, and F‐MR are defined similarly). The testing reward of the four one‐size‐fits‐all rules is estimated by setting $f_{1}=\pm 1$ and $f_{2}=\pm 1$ in $\hat{𝒱}(f)$, respectively.

^c^ Results are also reported in *mean(standard deviation)* format.

To sum up, the real data application results indicate that L1‐MRL continues to achieve overall better performance when applied to real data, as it simultaneously maintains treatment optimization while producing more reasonable, parsimonious, and clinically meaningful variable selection results that are consistent with domain evidence, compared to the other competing methods.

## 6 Discussion

Incorporating variable selection into optimal DTRs has drawn increasing attention in recent years, as it promotes the identification of unimportant feature variables with less contribution to treatment improvement, leading to more robust and parsimonious optimal rules. To address this challenge, we propose a machine learning‐based approach, namely L1‐MRL, by extending the existing MRL framework to simultaneously learn optimal rules and conduct variable selection. Benefiting from the simultaneous estimation property of MRL, the proposed framework can learn decision rules and perform variable selection across all stages at the same time. In contrast, existing methods, to the best of our knowledge, can only complete estimation and variable selection sequentially for each stage separately, thus suffering from accumulative estimation error and false discovery as reflected in our simulation studies. In terms of overall performance, our findings in this work show that L1‐MRL performs equally well as or better than regression‐based approaches when regression models are nearly correctly specified, and it outperforms competing methods when models are misspecified or in the real data application example. This makes L1‐MRL a particularly promising approach for optimal DTR problems with variable selection demands in practice, as the formulation of the optimal rule is usually unknown and prone to misspecification.

Apart from variable selection, assessing adverse risks or potential expenses caused by treatments and learning optimal rules under constraints has become another hot topic driven by real clinical demands. As one possible extension, L1‐MRL can be modified to incorporate variable selection under constraints by integrating these constraints into the optimization problem. However, as the objective of L1‐MRL is nonconvex, the formulation of constraints needs to be carefully designed to ensure numerical efficiency while preserving consistency and the oracle property. Extensions based on L1‐MRL or novel methods are expected to address the variable selection challenge when additional restrictions on the optimal rules must be satisfied at the same time. It is also worth noting that, apart from cross‐stage variable selection, the MRL framework can be extended to enforce various cross‐stage restrictions on decision rules by imposing different regularization terms, thanks to the simultaneous estimation property. One possible extension is to penalize the dissimilarity between recommended treatments in adjacent stages to avoid drastic treatment switches in a short time and reduce potential risks caused by unnecessary treatment adjustments. Future studies are expected to investigate and identify appropriate penalty terms that best reflect, such as treatment variation over time, to address various real‐world problems requiring different restrictions on decision rules across multiple stages.

As discussed in Remark 1, the performance guarantee of L1‐MRL relies on the known or consistent estimation of treatment assignment probabilities. Additionally, the performance of L1‐MRL requires a sufficiently large positive chance of observing the assigned treatment in the observed data, which is ensured by the introduction of Assumption 3 in this work. Our simulation findings indicate that L1‐MRL performs well when both requirements are satisfied. However, in many real‐world applications involving rare patients, consistent estimation and the positivity assumption may not hold. In particular, the performance of L1‐MRL will be negatively affected when the positivity assumption is violated, as the construction of the objective function relies on the inverse probability‐weighted estimator. Novel methods are still needed to address the variable selection challenge in optimal DTRs when either consistent estimation or the positivity assumption is violated. Lastly, due to its computational complexity, L1‐MRL is more suitable for applications with a moderate sample size of fewer than a thousand and becomes less efficient when applied to large‐scale datasets. Further numerical refinements to L1‐MRL or novel regression‐ and machine learning‐based approaches are still required to address the efficient variable selection challenge in optimal DTRs for large‐scale applications.

## Conflicts of Interest

The authors declare no conflicts of interest.

## Supporting information


**Data S1.** Supporting Information.

**Data S2.** Supporting Information.

## Data Availability

Data for the real application example (OSU EHR data) were acquired under a user agreement and are not publicly available. R codes to implement L1‐MRL and scripts for reproducing the simulation studies are available at https://github.com/mochuan‐liu/L1MRL‐Simulation.

## Appendix A Detail of Coordinate Descent DC Algorithm for Solving L1‐MRL

For convenience, we let

$$O_{i}=\frac{1}{n}\frac{Y_{i}}{\prod_{t=1}^{T}p(A_{it}|H_{it})}\;\text{or}\;O_{i}=\frac{1}{n}\frac{{\hat{Y}}_{i}}{\prod_{t=1}^{T}p(A_{it}|H_{it})}.$$

By subtracting and adding an additional term

$$\sum_{i=1}^{n}O_{i}^{-}\min(\psi (A_{i1}H_{i1}^{T}\theta_{1}⁄\eta_{n}),\ldots ,\psi (A_{iT}H_{iT}^{T}\theta_{T}⁄\eta_{n}))$$

in (4) or (5) in the main manuscript, one can show that the L1‐MRL problem is equivalent to maximizing

$$\begin{array}{ll} L(\theta ) & =\sum_{i=1}^{n}O_{i}\min(\psi (A_{i1}H_{i1}^{T}\theta_{1}⁄\eta_{n}),\ldots ,\psi (A_{iT}H_{iT}^{T}\theta_{T}⁄\eta_{n}))\\ & \;+\sum_{i=1}^{n}O_{i}^{-}\min(\psi (\left|H_{i1}^{T}\theta_{1}\right|⁄\eta_{n}),\ldots ,\psi (\left|H_{iT}^{T}\theta_{T}\right|⁄\eta_{n}))\\ & \;-\lambda_{n}\sum_{p=1}^{P}\sum_{t=1}^{T}\frac{\left|\theta_{t,p}\right|}{\sqrt{\sum_{s=1}^{T}{\hat{\tilde{\theta }}}_{s,p}^{2}}}\\ & =\sum_{i=1}^{n}\left|O_{i}\right|\min(A_{i1}H_{i1}^{T}\theta_{1}⁄\eta_{n},\ldots ,A_{iT}H_{iT}^{T}\theta_{T}⁄\eta_{n},d_{i})\\ & \;-\sum_{i=1}^{n}\left|O_{i}\right|\min(A_{i1}H_{i1}^{T}\theta_{1}⁄\eta_{n},\ldots ,A_{iT}H_{iT}^{T}\theta_{T}⁄\eta_{n},1-d_{i})\\ & \;+\sum_{i=1}^{n}O_{i}^{-}\min(\left|H_{i1}^{T}\theta_{1}\right|⁄\eta_{n},\ldots ,\left|H_{iT}^{T}\theta_{T}\right|⁄\eta_{n})-\lambda_{n}\sum_{p=1}^{P}\sum_{t=1}^{T}\frac{\left|\theta_{t,p}\right|}{\sqrt{\sum_{s=1}^{T}{\hat{\tilde{\theta }}}_{s,p}^{2}}}, \end{array}$$

where we use $\theta_{t,p}$ to denote $\beta_{t,p}$ to unify the notation, and recall that we assume the first P entries correspond to the coefficients that need to be penalized. In the previous equations, $d_{i}=𝕀(O_{i}\geq 0)$. To obtain the second equality, we have used the fact that

$$\min(\psi (x_{1}),\ldots ,\psi (x_{T}))=\min(x_{1},\ldots ,x_{T},1)-\min(x_{1},\ldots ,x_{T},0).$$

By reorganizing the objective function, we can equivalently minimize the new objective function

$$\begin{array}{ll} L^{\prime }(\theta ) & =\sum_{i=1}^{n}\left|O_{i}\right|\max(-A_{i1}H_{i1}^{T}\theta_{1}⁄\eta_{n},\ldots ,-A_{iT}H_{iT}^{T}\theta_{T}⁄\eta_{n},-d_{i})\\ & \;-\sum_{i=1}^{n}O_{i}^{-}\min(\left|H_{i1}^{T}\theta_{1}\right|⁄\eta_{n},\ldots ,\left|H_{iT}^{T}\theta_{T}\right|⁄\eta_{n})\\ & \;-\sum_{i=1}^{n}\left|O_{i}\right|\max(-A_{i1}H_{i1}^{T}\theta_{1}⁄\eta_{n},\ldots ,-A_{iT}H_{iT}^{T}\theta_{T}⁄\eta_{n},-(1-d_{i}))\\ & \;+\lambda_{n}\sum_{p=1}^{P}\sum_{t=1}^{T}\frac{\left|\theta_{t,p}\right|}{\sqrt{\sum_{s=1}^{T}{\hat{\tilde{\theta }}}_{s,p}^{2}}}. \end{array}$$

To obtain the solution for the L1‐MRL problem, we consider minimizing $L^{\prime }(\theta )$ via the coordinate descent. For convenience, we let $\theta_{t}^{\left(k\right)}=(\theta_{t,1}^{\left(k\right)},\ldots ,\theta_{t,K_{t}}^{\left(k\right)})$, $\theta_{t,-p}^{\left(k+1,k\right)}=(\theta_{t,1}^{\left(k+1\right)},\ldots ,\theta_{t,p-1}^{\left(k+1\right)},\theta_{t,p+1}^{\left(k\right)},\ldots ,\theta_{t,K_{t}}^{\left(k\right)})$ and $H_{it,-p}=(H_{it,1},\ldots ,H_{it,p-1},H_{it,p+1},\ldots ,H_{it,K_{t}})$, where $K_{t}$ denotes the number of unknown parameters at stage t. Given the current parameter vector $\left(\theta_{1,1}^{\left(k+1\right)},\ldots ,\theta_{t,p-1}^{\left(k+1\right)},\theta_{t,p}^{\left(k\right)},\ldots ,\theta_{T,K_{T}}^{\left(k\right)}\right)$, we update $\theta_{t,p}^{\left(k\right)}$ by fixing the remaining parameters as constants and solving the optimization problem

(A1)
$$\theta_{t,p}^{\left(k+1\right)}=\underset{\theta }{argmin}\;(S_{1}(\theta )-S_{2}(\theta ))$$

where

$$\begin{array}{ll} S_{1}(\theta ) & =\sum_{i=1}^{n}\left|O_{i}\right|\max(-A_{it}(H_{it,p}\theta +H_{it,-p}^{T}\theta_{t,-p}^{\left(k+1,k\right)})⁄\eta_{n},c_{it})\\ & \;+\sum_{i=1}^{n}O_{i}^{-}g_{2}((H_{it,p}\theta +H_{it,-p}^{T}\theta_{t,-p}^{\left(k+1,k\right)})⁄\eta_{n},c_{it}^{\prime \prime })+\gamma_{t,p}\lambda_{n}\frac{\left|\theta \right|}{\sqrt{\sum_{s=1}^{T}{\hat{\tilde{\theta }}}_{s,p}^{2}}},\\ S_{2}(\theta ) & =\sum_{i=1}^{n}\left|O_{i}\right|\max(-A_{it}(H_{it,p}\theta +H_{it,-p}^{T}\theta_{t,-p}^{\left(k+1,k\right)})⁄\eta_{n},c_{it^{\prime }})\\ & \;+\sum_{i=1}^{n}O_{i}^{-}g_{1}((H_{it,p}\theta +H_{it,-p}^{T}\theta_{t,-p}^{\left(k+1,k\right)})⁄\eta_{n}), \end{array}$$

with $\gamma_{t,p}=1$ if $\theta_{t,p}$ needs to be penalized and $\gamma_{t,p}=0$ otherwise, and

$$g_{1}(x)=|x|,\;g_{2}(x,c)=\max(-x-c,0)+\max(x-c,0),$$

$$\begin{array}{ll} c_{it} & =\max(-A_{i1}H_{i1}^{T}\theta_{1}^{\left(k+1\right)}⁄\eta_{n},\ldots ,-A_{i,t-1}H_{i,t-1}^{T}\theta_{t-1}^{\left(k+1\right)}⁄\eta_{n},\\ & \;-A_{i,t+1}H_{i,t+1}^{T}\theta_{t+1}^{\left(k\right)}⁄\eta_{n},\ldots ,-A_{iT}H_{iT}^{T}\theta_{T}^{\left(k\right)}⁄\eta_{n},-d_{i}),\\ c_{it}^{\prime } & =\max(-A_{i1}H_{i1}^{T}\theta_{1}^{\left(k+1\right)}⁄\eta_{n},\ldots ,-A_{i,t-1}H_{i,t-1}^{T}\theta_{t-1}^{\left(k+1\right)}⁄\eta_{n},\\ & \;-A_{i,t+1}H_{i,t+1}^{T}\theta_{t+1}^{\left(k\right)}⁄\eta_{n},\ldots ,-A_{iT}H_{iT}^{T}\theta_{T}^{\left(k\right)}⁄\eta_{n},-(1-d_{i})),\\ c_{it}^{\prime } & =\max\left(\left|H_{i1}^{T}\theta_{1}^{\left(k+1\right)}\right|⁄\eta_{n},\ldots ,\left|H_{i,t-1}^{T}\theta_{t-1}^{\left(k+1\right)}\right|⁄\eta_{n},\right.\\ & \;\left.\left|H_{i,t+1}^{T}\theta_{t+1}^{\left(k\right)}\right|⁄\eta_{n},\ldots ,\left|H_{iT}^{T}\theta_{T}^{\left(k\right)}\right|⁄\eta_{n}\right). \end{array}$$

Note that both $S_{1}$ and $S_{2}$ are convex function of θ. Therefore, (A1) can be solved by applying the DC algorithm [26], where we iteratively solve

(A2)
$$\theta^{\left(s+1\right)}=\min_{\theta }\;\left(S_{1}(\theta )-\frac{\partial S_{2}}{\partial \theta }(\theta^{\left(s\right)})(\theta -\theta^{\left(s\right)})\right)$$

until convergence, starting from $\theta^{\left(0\right)}=\theta_{t,p}^{\left(k\right)}$. To further reduce the computational complexity, we approximate the subgradient $\frac{\partial S_{2}}{\partial \theta }(\theta )$ by

$$\begin{array}{cc} \frac{\partial {\tilde{S}}_{2}}{\partial \theta }(\theta ) & =\sum_{i=1}^{n}\frac{1}{\eta_{n}}\left|O_{i}\right|(-A_{it}H_{it,p})\frac{e^{-A_{it}(H_{it,p}\theta +H_{it,-p}^{T}\theta_{t,-p}^{\left(k+1,k\right)})⁄\eta_{n}}}{e^{-A_{it}(H_{it,p}\theta +H_{it,-p}^{T}\theta_{t,-p}^{\left(k+1,k\right)})⁄\eta_{n}}+e^{c_{it}^{\prime }}}\\ & \;+\sum_{i=1}^{n}\frac{1}{\eta_{n}}O_{i}^{-}H_{it,p}\text{sign}(H_{it,p}\theta +H_{it,-p}^{T}\theta_{t,-p}^{\left(k+1,k\right)}) \end{array}$$

using the smoothing technique from Nesterov [44]. For fixed t and p, the optimization of (A2) with respect to θ becomes a minimization problem with respect to a piecewise linear function, which can be efficiently solved by calculating the derivatives at each endpoint. For each t and p, we update $\theta_{t,p}^{\left(k\right)}$ until the DC procedure (A2) converges, and the coordinate descent algorithm terminates when $\theta^{\left(k\right)}=\{\theta_{1}^{\left(k\right)},\ldots ,\theta_{T}^{\left(k\right)}\}$ converges.

## Appendix B Proof of Theorem 1

We continue using the notation from Appendix A and complete the proof by first verifying the lemma below.

Lemma 1
*For any compact subset* ${𝒳}_{t}\subset {\mathbb{R}}^{d_{t}}$ *and* B>0 *, let* ${\left\{X_{t}\right\}}_{t=1}^{T}$ *be* T *random vectors defined on spaces* ${\left\{{𝒳}_{t}\right\}}_{t=1}^{T}$ *. Define*

$$\begin{array}{ll} 𝒲 & =\left\{\min(\psi (X_{1}^{T}\theta_{1}),\ldots ,\psi (X_{T}^{T}\theta_{T})):\theta_{t}\in {\mathbb{R}}^{d_{t}},\;{\left\|\theta_{t}\right\|}_{\infty }\leq B,\;t=1,\ldots ,T\right\}, \end{array}$$

*then for any* δ>0 *, we have*

$$P(\underset{w\in 𝒲}{\sup}|{\mathbb{P}}_{n}[w]-E[w]|\geq c\frac{\sqrt{\sum_{t}d_{t}\log(BTd_{t})}}{\sqrt{n}}+\delta )\leq c^{\prime }e^{-n\delta^{2}},$$

*where* c *and* $c^{\prime }$ *are constants that do not depend on the sample size* n.

Without loss of generality, we assume that ${𝒳}_{t}$ is the unit ball of ${\mathbb{R}}^{d_{t}}$ for t=1,…,T. Let ℬ denote the centers of an $\frac{\epsilon }{d_{t}T}$ covering of the interval [−B,B] under the Euclidean distance. For an arbitrary w∈𝒲 associated with coefficients ${\left\{\theta_{t}\right\}}_{t=1}^{T}$, by definition, we can find centers $b_{t}\in {ℬ}^{d_{t}}$ for t=1,…,T such that

$${\left\|b_{t}-\theta_{t}\right\|}_{\infty }\leq \frac{\epsilon }{d_{t}T}.$$

Using the fact that for any $\left(x_{1},\ldots ,x_{T}\right)$ and $\left(x_{1}^{\prime },\ldots ,x_{T}^{\prime }\right)$,

(B1)
$$\begin{array}{ll} & \left|\min(\psi (x_{1}),\ldots ,\psi (x_{T}))-\min(\psi (x_{1}^{\prime }),\ldots ,\psi (x_{T}^{\prime }))\right|\leq \sum_{t=1}^{T}\left|x_{t}-x_{t}^{\prime }\right| \end{array}$$

we further have

$$\begin{array}{ll} & \left|w-\min(\psi (X_{1}^{T}b_{1}),\ldots ,\psi (X_{T}^{T}b_{T}))\right|\leq \sum_{t=1}^{T}d_{t}{\left\|b_{t}-\theta_{t}\right\|}_{\infty }\leq \epsilon , \end{array}$$

where the first inequality follows from the fact that ψ(x) is a 1‐Lipschitz function. Since

$$𝒩\left([-B,B],\epsilon ;\;{\left\|\;\cdot \;\right\|}_{\infty }\right)\leq \frac{B}{\epsilon },$$

the previous inequality implies that

$$𝒩\left(𝒲,\epsilon ;\;{\left\|\;\cdot \;\right\|}_{\infty }\right)\leq \prod_{t=1}^{T}{\left(\frac{BTd_{t}}{\epsilon }\right)}^{d_{t}}$$

Now, we show that the stated concentration inequality holds. Using Theorem 4.10 from [45], we have

$$P(\underset{w\in 𝒲}{\sup}|{\mathbb{P}}_{n}[w]-E[w]|\geq 2{\text{Rad}}_{n}(𝒲)+\delta )\geq ce^{-n\delta^{2}}$$

holds for any δ>0, where ${\text{Rad}}_{n}(𝒲)$ denotes the Rademacher complexity of 𝒲, defined as

$${\text{Rad}}_{n}(𝒲)=\underset{w\in 𝒲}{\sup}E_{X}\left[E_{\epsilon }\left[\left|\frac{1}{n}\sum_{i=1}^{n}\epsilon_{i}w(X_{i})\right|\right]\right],\;P(\epsilon_{i}=\pm 1)=0.5.$$

Here, we abuse notation and use $w(X_{i})$ to denote the i.i.d. replication of w evaluated at $X_{i}$. Using Example 5.24 from Wainwright et al. [45], we also have

$$E_{\epsilon }\left[\left|\frac{1}{n}\sum_{i=1}^{n}\epsilon_{i}w(X_{i})\right|\right]\leq \frac{24}{\sqrt{n}}\int_{0}^{2}\sqrt{\log 𝒩(𝒲,\epsilon ;\;{\left\|\;\cdot \;\right\|}_{{\mathbb{P}}_{n}})}d\epsilon ,$$

where $\|\cdot {\|}_{{\mathbb{P}}_{n}}$ denotes the empirical $L_{2}$‐norm, defined as

$${\left\|w_{1}-w_{2}\right\|}_{{\mathbb{P}}_{n}}^{2}=\frac{1}{n}\sum_{i=1}^{n}(w_{1}(x_{i})-w_{2}{\left(x_{i})\right)}^{2}.$$

Since ${\left\|w_{1}-w_{2}\right\|}_{{\mathbb{P}}_{n}}\leq {\left\|w_{1}-w_{2}\right\|}_{\infty }$, we have

$$𝒩(𝒲,\epsilon ;\;{\left\|\cdot \right\|}_{{\mathbb{P}}_{n}})\leq 𝒩(𝒲,\epsilon ;\;{\left\|\cdot \right\|}_{\infty }),$$

and consequently

$$E_{\epsilon }\left[\left|\frac{1}{n}\sum_{i=1}^{n}\epsilon_{i}w(X_{i})\right|\right]\leq c\frac{\sqrt{\sum_{t}d_{t}\log(BTd_{t})}}{\sqrt{n}}.$$

The inequality is verified by taking the expectation with respect to X.

We now complete the proof of Theorem 1 using Lemma B.1.

We first prove the first part of the theorem and show that ${\left\|\hat{\theta }-\theta^{*}\right\|}_{2}=O_{p}(n^{-\frac{1}{4}+\frac{\xi }{2}})$. For convenience, we define

$$\begin{array}{ll} {ℒ}_{1}(\theta ) & =-E\left[Y^{+}\frac{\min(\psi (A_{1}H_{1}^{T}\theta_{1}),\ldots ,\psi (A_{T}H_{T}^{T}\theta_{T}))}{\prod_{t=1}^{T}p(A_{t}|H_{t})}\right],\\ {ℒ}_{2}(\theta ) & =-E\left[\sum_{\overset{(a_{1},\ldots ,a_{T})\in {\left\{-1,+1\right\}}^{T}}{\exists t\;such\;that\;a_{t}\neq A_{t}}}Y^{-}\frac{\min(\psi (a_{1}H_{1}^{T}\theta_{1}),\ldots ,\psi (a_{T}H_{T}^{T}\theta_{T}))}{\prod_{t=1}^{T}p(A_{t}|H_{t})}\right], \end{array}$$

and use ${𝒬}_{1}(\theta )$ and ${𝒬}_{2}(\theta )$ to denote the empirical versions of ${ℒ}_{1}(\theta )$ and ${ℒ}_{2}(\theta )$, respectively. Without loss of generality, we assume that the q+1 to P entries of ${\left\{\theta^{*}\right\}}_{t=1}^{T}$ correspond to the coefficients of the variables in ${\left\{O_{t}\right\}}_{t=1}^{T}$ that are insignificant across all stages. Let 𝒬(θ) denote the objective function of the minimization equivalence of (4). By definition, we have

$$𝒬(\theta )={𝒬}_{1}(\theta )+{𝒬}_{2}(\theta )+\sum_{t,p}\lambda_{n}\omega_{p,\gamma }\left|\theta_{t,p}\right|$$

Let $\alpha_{n}=\lambda_{n}+n^{-\frac{1}{4}+\frac{\xi }{2}}$, which has order $O_{p}(n^{-\frac{1}{4}+\frac{\xi }{2}})$ since $\lambda_{n}n^{\frac{1}{4}-\frac{\xi }{2}}=o(1)$. Our goal is to show that

$$Q(\theta^{*}+\alpha_{n}\delta )>Q(\theta^{*})$$

holds for any ${\left\|\delta \right\|}_{2}=C$ for some sufficiently large C that does not depend on the sample size n, with probability 1 as n goes to infinity. Here, δ denotes a vector of the same length as $\theta^{*}$. To show this, we first note that

$$\begin{array}{ll} & Q(\theta^{*}+\alpha_{n}\delta )-Q(\theta^{*})\\ & \geq -|{ℒ}_{1}(\theta^{*}+\alpha_{n}\delta )-{𝒬}_{1}(\theta^{*}+\alpha_{n}\delta )|-|{ℒ}_{2}(\theta^{*}+\alpha_{n}\delta )-{𝒬}_{2}(\theta^{*}+\alpha_{n}\delta )|\\ & \;-|{ℒ}_{1}(\theta^{*})-{𝒬}_{1}(\theta^{*})|-|{ℒ}_{2}(\theta^{*})-{𝒬}_{2}(\theta^{*})|\\ & \;+\underset{I}{\underbrace{ℒ(\theta^{*}+\alpha_{n}\delta )-ℒ(\theta^{*})+\sum_{t,p}\lambda_{n}\omega_{p,\gamma }\left|\theta_{t,p}^{*}+\alpha_{n}\delta_{t,p}\right|-\sum_{t,p}\lambda_{n}\omega_{p,\gamma }\left|\theta_{t,p}^{*}\right|}}. \end{array}$$

By the Taylor expansion of $ℒ(\theta^{*}+\alpha_{n}\delta )$ at $\theta^{*}$ and Assumption 4, term I on the right‐hand side of the inequality above is lower bounded by

(B2)
$$I\geq c_{1}\alpha_{n}^{2}\delta^{T}\frac{\partial^{2}ℒ}{{\left(\partial \theta \right)}^{2}}(\theta^{*})\delta -c_{2}\lambda_{n}\alpha_{n}+o_{p}(\alpha_{n}^{2})$$

for some positive constants $c_{1}$ and $c_{2}$ that do not depend on the sample size n and constant C. Since $\lambda_{n}n^{\frac{1}{4}-\frac{\xi }{2}}=o(1)$ and $\frac{\partial^{2}ℒ}{{\left(\partial \theta \right)}^{2}}(\theta^{*})$ is positive definite, we can choose a sufficiently large C such that $c_{1}\alpha_{n}^{2}\delta^{T}\nabla^{2}ℒ(\theta^{*})\delta$ dominates the remaining terms for any $\|\delta {\|}_{2}=C$. Hence, the term I has order $\alpha_{n}^{2}=n^{-\frac{1}{2}+\xi }$, up to a positive constant that does not depend on n, for any sufficiently large n. On the other hand, under Assumption 4, Lemma B. 1 implies that

$$\begin{array}{ll} & |{ℒ}_{1}(\theta^{*}+\alpha_{n}\delta )-{𝒬}_{1}(\theta^{*}+\alpha_{n}\delta )|=O_{p}(n^{-\frac{1}{2}}),\;\;\\ & |{ℒ}_{2}(\theta^{*}+\alpha_{n}\delta )-{𝒬}_{2}(\theta^{*}+\alpha_{n}\delta )|=O_{p}(n^{-\frac{1}{2}}),\;\\ & |{ℒ}_{1}(\theta^{*})-{𝒬}_{1}(\theta^{*})|=O_{p}(n^{-\frac{1}{2}}),\;|{ℒ}_{2}(\theta^{*})-{𝒬}_{2}(\theta^{*})|=O_{p}(n^{-\frac{1}{2}}).\; \end{array}$$

Combining this with (B2), we obtain

$$Q(\theta^{*}+\alpha_{n}\delta )-Q(\theta^{*})>O_{p}(n^{-\frac{1}{2}})+I=I(1+o_{p}(1))>0$$

for sufficiently large n. The discussion above indicates that for any ϵ>0

$$P\left(\underset{{\left\|\delta \right\|}_{2}=C}{\inf}Q(\theta^{*}+\alpha_{n}\delta )\geq Q(\theta^{*})\right)\geq 1-\epsilon ,$$

for any sufficiently large n. This further implies that there always exists a local minimum $\hat{\theta }$ such that ${\left\|\hat{\theta }-\theta^{*}\right\|}_{2}=O_{p}(n^{-\frac{1}{4}+\frac{\xi }{2}})$. This completes the proof of the first part of the theorem. To prove the second part of the oracle property, for simplicity, we write an arbitrary vector δ of length $\theta^{*}$ as $\delta =(\delta^{1},\delta^{2})$, where $\delta^{2}$ denotes the concatenation of the q+1 to P entries of ${\left\{\delta_{t}\right\}}_{t=1}^{T}$, corresponding to the indices of $\left\{\theta_{t,p}^{*}\right\}$ that are equal to 0 across all stages. Now, let $\alpha_{n}=n^{-\frac{1}{4}+\frac{\xi }{2}}$ and define $G_{n}(\delta )=\sum_{i=1}^{n}l_{i}(\delta )$, where

$$\begin{array}{ll} & l_{i}(\delta )\\ & =-Y_{i}^{+}\frac{\min(\psi (A_{i1}H_{i1}(\theta_{1}^{*}+\alpha_{n}\delta_{1})),\ldots ,\psi (A_{iT}H_{iT}(\theta_{T}^{*}+\alpha_{n}\delta_{T})))}{\prod_{t=1}^{T}p(A_{it}|H_{it})}\\ & \;\;-\sum_{\overset{(a_{1},\ldots ,a_{T})\in {\left\{-1,+1\right\}}^{T}}{\exists t\;such\;that\;a_{t}\neq A_{it}}}Y_{i}^{-}\frac{\min(\psi (a_{1}H_{i1}(\theta_{1}^{*}+\alpha_{n}\delta_{1})),\ldots ,\psi (a_{T}H_{iT}(\theta_{T}^{*}+\alpha_{n}\delta_{T})))}{\prod_{t=1}^{T}p(A_{it}|H_{it})}\\ & \;\;+Y_{i}^{+}\frac{\min(\psi (A_{i1}H_{i1}\theta_{1}^{*}),\ldots ,\psi (A_{iT}H_{iT}\theta_{T}^{*}))}{\prod_{t=1}^{T}p(A_{it}|H_{it})}\\ & \;\;+\sum_{\overset{(a_{1},\ldots ,a_{T})\in {\left\{-1,+1\right\}}^{T}}{\exists t\;such\;that\;a_{t}\neq A_{it}}}Y_{i}^{-}\frac{\min(\psi (a_{1}H_{i1}\theta_{1}^{*}),\ldots ,\psi (a_{T}H_{iT}\theta_{T}^{*}))}{\prod_{t=1}^{T}p(A_{it}|H_{it})}. \end{array}$$

We also recall that $\rho_{\lambda_{n},\gamma }(\delta )=\lambda_{n}\sum_{t,p}\omega_{p,\gamma }\left|\delta_{t,p}\right|$. Following the same proof scheme as Theorem 3 of Wu et al. [46], it is sufficient to show that for any $\delta^{1}=(\delta^{11},\delta^{12})$ and $\delta^{2}=(\delta^{21},0)$ such that $\left\|\delta^{11}\right\|\leq C$, $\left\|\delta^{12}\right\|\leq C$, and $0<\left\|\delta^{21}\right\|\leq C$ for some fixed constant C>0, we have

$$G_{n}(\delta^{2})+n\rho_{\lambda_{n},\gamma }(\delta^{2})-[G_{n}(\delta^{1})+n\rho_{\lambda_{n},\gamma }(\delta^{1})]\to -\infty$$

with probability 1 as n goes to infinity. To complete the proof, first note that

$$G_{n}(\delta )=\underset{I_{1}}{\underbrace{E[G_{n}(\delta )]}}+\underset{I_{2}}{\underbrace{G_{n}(\delta )-E[G_{n}(\delta )]}}.$$

By the Taylor expansion of ℒ(θ+αδ) at $\theta^{*}$, for $I_{1}$, we have

$$I_{1}\;=\;\overset{n}{\underset{i=1}{\sum }}E[l_{i}(\delta )]\;=\;C^{\prime }n\alpha_{n}^{2}\delta^{T}\frac{\partial^{2}ℒ}{{\left(\partial \theta \right)}^{2}}(\theta^{*})\delta +o_{p}(n\alpha_{n}^{2})=O_{p}(n\alpha_{n}^{2}).$$

For $I_{2}$, we have

$$\begin{array}{ll} & E[(G_{n}(\delta )-E[G_{n}{\left(\delta )]\right)}^{2}]=\sum_{i=1}^{n}E[(l_{i}(\delta )-E[l_{i}{\left(\delta )]\right)}^{2}]\leq \sum_{i=1}^{n}E[l_{i}{\left(\delta \right)}^{2}]. \end{array}$$

Applying (B1) and noting that ψ(·) s a 1‐Lipschitz function, for each i we have

$$\begin{array}{rl} & l_{i}(\delta )\\ & =-Y_{i}^{+}\frac{\min(\psi (A_{i1}H_{i1}(\theta_{1}^{*}+\alpha_{n}\delta_{1})),\ldots ,\psi (A_{iT}H_{iT}(\theta_{T}^{*}+\alpha_{n}\delta_{T})))}{\prod_{t=1}^{T}p(A_{it}|H_{it})}\\ & \;+Y_{i}^{+}\frac{\min(\psi (A_{i1}H_{i1}\theta_{1}^{*}),\ldots ,\psi (A_{iT}H_{iT}\theta_{T}^{*}))}{\prod_{t=1}^{T}p(A_{it}|H_{it})}\\ & \;-\sum_{\overset{(a_{1},\ldots ,a_{T})\in {\left\{-1,+1\right\}}^{T}}{\exists t\;such\;that\;a_{t}\neq A_{it}}}Y_{i}^{-}\frac{\min(\psi (a_{1}H_{i1}(\theta_{1}^{*}+\alpha_{n}\delta_{1})),\ldots ,\psi (a_{T}H_{iT}(\theta_{T}^{*}+\alpha_{n}\delta_{T})))}{\prod_{t=1}^{T}p(A_{it}|H_{it})}\\ & \;+\sum_{\overset{(a_{1},\ldots ,a_{T})\in {\left\{-1,+1\right\}}^{T}}{\exists t\;such\;that\;a_{t}\neq A_{it}}}Y_{i}^{-}\frac{\min(\psi (a_{1}H_{i1}\theta_{1}^{*}),\ldots ,\psi (a_{T}H_{iT}\theta_{T}^{*}))}{\prod_{t=1}^{T}p(A_{it}|H_{it})}\\ & \leq C^{\prime }\sum_{t=1}^{T}\sum_{a_{t}\in \{-1,+1\}}\left|\psi (a_{t}H_{it}(\theta_{t}^{*}+\alpha_{n}\delta_{t}))-\psi (a_{t}H_{it}\theta_{t}^{*})\right|\\ & \leq C^{\prime }\alpha_{n}. \end{array}$$

Hence, we have $E[{\left(G_{n}(\delta )-E[G_{n}(\delta )]\right)}^{2}]=O(n\alpha^{2})$, which further implies that $I_{2}=O_{p}\left(n^{1+\frac{\xi^{\prime }-\xi }{2}}\alpha_{n}^{2}\right)$ for any $\xi <\xi^{\prime }$. Consequently, by setting δ to $\delta^{1}$ and $\delta^{2}$, we obtain

$$G_{n}(\delta^{2})-G_{n}(\delta^{1})=I_{1}+I_{2}=O_{p}\left(n^{1+\frac{\xi^{\prime }-\xi }{2}}\alpha_{n}^{2}\right).$$

On the other hand

$$n\rho_{\lambda_{n},\gamma }(\delta^{2})-n\rho_{\lambda_{n},\gamma }(\delta^{1})=\underset{I_{3}}{\underbrace{n\{\rho_{\lambda_{n},\gamma }((\delta^{21},0))-\rho_{\lambda_{n},\gamma }((\delta^{11},0))\}}}-\underset{I_{4}}{\underbrace{n\rho_{\lambda_{n},\gamma }((0,\delta^{12}))}},$$

where

$$I_{3}\leq \sum_{t=1,\ldots ,T,\;p\leq q}n\lambda_{n}\alpha_{n}\omega_{p}\left|\delta_{t,p}^{21}-\delta_{t,p}^{11}\right|\leq O_{p}(n\lambda_{n}\alpha_{n})=o_{p}(n\alpha_{n}^{2})$$

and

$$\begin{array}{ll} I_{4} & =C^{\prime }n\sum_{t=1,\ldots ,T,p>q}\lambda_{n}\alpha_{n}\frac{\left|\delta_{t,p}^{12}\right|}{{\left(\sum_{t=1}^{T}{\left|{\hat{\tilde{\theta }}}_{t,p}\right|}^{2}\right)}^{\frac{\gamma }{2}}}\\ & =C^{\prime }n\sum_{t=1,\ldots ,T,p>q}\lambda_{n}\alpha_{n}\frac{\left|\delta_{t,p}^{12}\right|}{{\left(\sqrt{n}\sqrt{\sum_{t=1}^{T}{\left|{\hat{\tilde{\theta }}}_{t,p}\right|}^{2}}\right)}^{\gamma }}n^{\frac{\gamma }{2}}\\ & =C^{\prime \prime }n^{1+\frac{\gamma }{2}}\lambda_{n}\alpha_{n}^{2} \end{array}$$

for appropriate γ. Hence, when $\lambda_{n}n^{\frac{1}{4}-\frac{\xi^{\prime }}{2}+\frac{\gamma }{2}}\to \infty$, $I_{3}-I_{4}$ will have a higher order and dominate $I_{1}+I_{2}$, which goes to negative infinity as n goes to infinity. This completes the proof of the second part of the theorem.

## References

[1] B. Chakraborty and E. E. Moodie , Statistical Methods for Dynamic Treatment Regimes: Reinforcement Learning, Causal Inference, and Personalized Medicine, 1st ed. (Springer, 2013).

[2] S. A. Murphy , “Optimal Dynamic Treatment Regimes,” Journal of the Royal Statistical Society: Series B 65, no. 2 (2003): 331–355.

[3] J. M. Robins , “Optimal Structural Nested Models for Optimal Sequential Decisions,” in Proceedings of the Second Seattle Symposium in Biostatistics: Analysis of Correlated Data, 1st ed., ed. D. Lin and P. Heagert (Springer, 2004).

[4] M. Qian and S. A. Murphy , “Performance Guarantees for Individualized Treatment Rules,” Annals of Statistics 39, no. 2 (2011): 1180–1210. 21666835 10.1214/10-AOS864 PMC3110016

[5] M. P. Wallace and E. E. M. Moodie , “Doubly‐Robust Dynamic Treatment Regimen Estimation via Weighted Least Squares,” Biometrics 71, no. 3 (2015): 636–644. 25854539 10.1111/biom.12306

[6] R. Henderson , P. Ansell , and D. Alshibani , “Regret‐Regression for Optimal Dynamic Treatment Regimes,” Biometrics 66, no. 4 (2010): 1192–1201. 20002404 10.1111/j.1541-0420.2009.01368.x

[7] D. Almirall , T. Ten Have , and S. A. Murphy , “Structural Nested Mean Models for Assessing Time‐Varying Effect Moderation,” Biometrics 66, no. 1 (2010): 131–139. 19397586 10.1111/j.1541-0420.2009.01238.x PMC2875310

[8] Y. Zhao , D. Zeng , E. B. Laber , and M. R. Kosorok , “New Statistical Learning Methods for Estimating Optimal Dynamic Treatment Regimes,” Journal of the American Statistical Association 110, no. 510 (2015): 583–598. 26236062 10.1080/01621459.2014.937488 PMC4517946

[9] Y. Zhang , E. B. Laber , A. Tsiatis , and M. Davidian , “Using Decision Lists to Construct Interpretable and Parsimonious Treatment Regimes,” Biometrics 71, no. 4 (2015): 895–904. 26193819 10.1111/biom.12354 PMC4715597

[10] E. B. Laber and Y. Zhao , “Tree‐Based Methods for Individualized Treatment Regimes,” Biometrika 102, no. 3 (2015): 501–514. 26893526 10.1093/biomet/asv028 PMC4755313

[11] Y. Tao , L. Wang , and D. Almirall , “Tree‐Based Reinforcement Learning for Estimating Optimal Dynamic Treatment Regimes,” Annals of Applied Statistics 12, no. 3 (2018): 1914–1938. 30984321 10.1214/18-AOAS1137 PMC6457899

[12] X. Qiu and Y. Wang , “Composite Interaction Tree for Simultaneous Learning of Optimal Individualized Treatment Rules and Subgroups,” Statistics in Medicine 38, no. 14 (2019): 2632–2651. 30891797 10.1002/sim.8105 PMC8548070

[13] Y. Liu , Y. Wang , M. R. Kosorok , Y. Zhao , and D. Zeng , “Augmented Outcome‐Weighted Learning for Estimating Optimal Dynamic Treatment Regimens,” Statistics in Medicine 37, no. 26 (2018): 3776–3788. 29873099 10.1002/sim.7844 PMC6191367

[14] P. M. Bossuyt and T. Parvin , “Evaluating Biomarkers for Guiding Treatment Decisions,” Ejifcc 26, no. 1 (2015): 63–70. 27683482 PMC4975224

[15] L. Gunter , J. Zhu , and S. A. Murphy , “Variable Selection for Qualitative Interactions in Personalized Medicine While Controlling the Family‐Wise Error Rate,” Journal of Biopharmaceutical Statistics 21, no. 6 (2011): 1063–1078. 22023676 10.1080/10543406.2011.608052 PMC3396291

[16] R. Song , W. Wang , D. Zeng , and M. R. Kosorok , “Penalized Q‐Learning for Dynamic Treatment Regimens,” Statistica Sinica 25, no. 3 (2015): 901–920. 26257504 10.5705/ss.2012.364 PMC4526274

[17] C. Shi , A. Fan , R. Song , and W. Lu , “High‐Dimensional A‐Learning for Optimal Dynamic Treatment Regimes,” Annals of Statistics 46, no. 3 (2018): 925–957. 29805186 10.1214/17-AOS1570 PMC5966293

[18] Z. Bian , E. E. M. Moodie , S. M. Shortreed , and S. Bhatnagar , “Variable Selection in Regression‐Based Estimation of Dynamic Treatment Regimes,” Biometrics 79, no. 2 (2023): 988–999. 34837380 10.1111/biom.13608 PMC11350356

[19] W. Guo , X. Zhou , and S. Ma , “Estimation of Optimal Individualized Treatment Rules Using a Covariate‐Specific Treatment Effect Curve With High‐Dimensional Covariates,” Journal of the American Statistical Association 116, no. 533 (2021): 309–321.

[20] W. Lu , H. H. Zhang , and D. Zeng , “Variable Selection for Optimal Treatment Decision,” Statistical Methods in Medical Research 22, no. 5 (2013): 493–504. 22116341 10.1177/0962280211428383 PMC3303960

[21] R. Song , M. R. Kosorok , D. Zeng , Y. Zhao , E. B. Laber , and M. Yuan , “On Sparse Representation for Optimal Individualized Treatment Selection With Penalized Outcome Weighted Learning,” Stat 4, no. 1 (2015): 59–68. 25883393 10.1002/sta4.78 PMC4394905

[22] X. Li , S. Xie , D. Zeng , and Y. Wang , “Efficient l0‐Norm Feature Selection Based on Augmented and Penalized Minimization,” Statistics in Medicine 37, no. 3 (2018): 473–486. 29082539 10.1002/sim.7526 PMC5768461

[23] Y. He , S. Kim , M. O. Kim , W. Saber , and K. W. Ahn , “Optimal Treatment Regimes for Competing Risk Data Using Doubly Robust Outcome Weighted Learning With bi‐Level Variable Selection,” Computational Statistics & Data Analysis 158 (2021): 107167. 33994608 10.1016/j.csda.2021.107167 PMC8117077

[24] Y. Huang , “Identifying Optimal Biomarker Combinations for Treatment Selection Through Randomized Controlled Trials,” Clinical Trials 12, no. 4 (2015): 348–356. 25948620 10.1177/1740774515580126 PMC4506270

[25] M. Liu , Y. Wang , H. Fu , and D. Zeng , “Controlling Cumulative Adverse Risk in Learning Optimal Dynamic Treatment Regimens,” Journal of the American Statistical Association 119, no. 548 (2024): 2622–2633. 10.1080/01621459.2023.2270637 PMC12323820 40766209

[26] T. Pham Dinh and H. A. Le Thi , “Convex Analysis Approach to DC Programming: Theory, Algorithms and Applications,” Acta Mathematica Vietnamica 22, no. 1 (1997): 289–355.

[27] E. B. Laber , D. J. Lizotte , M. Qian , W. E. Pelham , and S. A. Murphy , “Dynamic Treatment Regimes: Technical Challenges and Applications,” Electronic Journal of Statistics 8, no. 1 (2014): 1225–1272. 25356091 10.1214/14-ejs920 PMC4209714

[28] N. Deliu and B. Chakraborty , “Dynamic Treatment Regimes for Optimizing Healthcare,” in The Elements of Joint Learning and Optimization in Operations Management, 1st ed., ed. X. Chen , S. Jasin , and C. Shi (Springer, 2022).

[29] M. A. Hernán and J. M. Robins , Causal Inference: What if (Taylor and Francis, 2024).

[30] D. B. Rubin , “Discussion of “Randomized Analysis of Experimental Data: The Fisher Randomization Test” by D. Basu,” Journal of the American Statistical Association 75 (1980): 591–593.

[31] J. M. Robins , “Causal Inference From Complex Longitudinal Data,” in Latent Variable Modeling and Applications to Causality: Lecture Notes in Statistics, 1st ed., ed. M. Berkane (Springer, 1997), 69–117.

[32] S. A. Murphy , “An Experimental Design for the Development of Adaptive Treatment Strategies,” Statistics in Medicine 24, no. 10 (2005): 1455–1481. 15586395 10.1002/sim.2022

[33] X. Huang , L. Shi , and J. A. Suykens , “Ramp Loss Linear Programming Support Vector Machine,” Journal of Machine Learning Research 15, no. 1 (2014): 2185–2211.

[34] H. Bozdogan , “Model Selection and Akaike's Information Criterion (AIC): The General Theory and Its Analytical Extensions,” Psychometrika 52, no. 3 (1987): 345–370.

[35] J. Fan and R. Li , “Variable Selection via Nonconcave Penalized Likelihood and Its Oracle Properties,” Journal of the American Statistical Association 96, no. 456 (2001): 1348–1360.

[36] C. Zhang , “Nearly Unbiased Variable Selection Under Minimax Concave Penalty,” Annals of Statistics 38, no. 2 (2010): 894–942.

[37] E. Candes and T. Tao , “The Dantzig Selector: Statistical Estimation When p Is Much Larger Than n ,” Annals of Statistics 35, no. 6 (2007): 2313–2351.

[38] C. Cortes and V. Vapnik , “Support‐Vector Networks,” Machine Learning 20, no. 3 (1995): 273–297.

[39] American Diabetes Association , “Standards of Care in Diabetes‐2023 Abridged for Primary Care Providers,” Clinical Diabetes 41, no. 1 (2022): 4–31. 36714254 10.2337/cd23-as01 PMC9845083

[40] American Diabetes Association , “Glycemic Goals and Hypoglycemia: Standards of Care in Diabetes—2025,” Diabetes Care 48, no. Supplement_1 (2025): S128–S145. 39651981 10.2337/dc25-S006 PMC11635034

[41] R. A. McPherson , M. R. Pincus , and J. B. Henry , Henry's Clinical Diagnosis and Management by Laboratory Methods, 24th ed. (Elsevier, 2021).

[42] American Diabetes Association , “Pharmacologic Approaches to Glycemic Treatment: Standards of Care in Diabetes—2025,” Diabetes Care 48, no. Supplement_1 (2025): S181–S206. 39651989 10.2337/dc25-S009 PMC11635045

[43] H. Yki‐Järvinen , L. Ryysy , M. Kauppila , et al., “Effect of Obesity on the Response to Insulin Therapy in Noninsulin‐Dependent Diabetes Mellitus,” Journal of Clinical Endocrinology & Metabolism 82, no. 12 (1997): 4037–4043. 9398709 10.1210/jcem.82.12.4460

[44] Y. Nesterov , “Smooth Minimization of Non‐Smooth Functions,” Mathematical Programming 103, no. 1 (2005): 127–152.

[45] M. J. Wainwright , High‐Dimensional Statistics: A Non‐Asymptotic Viewpoint, 1st ed. (Cambridge University Press, 2019).

[46] Y. Wu and Y. Liu , “Variable Selection in Quantile Regression,” Statistica Sinica 19, no. 2 (2009): 801–817.

