# Supplementary material for: Simultaneous Feature Selection for Optimal Dynamic Treatment Regimens
Source: Stat Med. 2025 Jul 15;44(15-17):e70169. doi: 10.1002/sim.70169 (PMC12261976; doi:10.1002/sim.70169)
Supplement: Supplementary file 1 — Data S1. Supporting Information. [file SIM-44-0-s001.pdf]

# Supplemental Materials for "Simultaneous Feature Selection for Optimal Dynamic Treatment Regimens"

## S.1 Additional simulation study with a different number of important variables in each stage

In this section, we conduct an additional simulation study with $T = 2$ to investigate the performance of the proposed method when the number of important variables differs in each stage. In this study, feature variables $H_1 = (Z_1, ..., Z_{12}, X_{11}, X_{21}, X_{31})$ and $H_2 = (Z_1, ..., Z_{12}, X_{12}, X_{22}, X_{32})$ are generated in the same way as in Section 4 of the main paper. Treatments $(A_1, A_2)$ are assigned according to the regression models:

$$\text{logit } P(A_1 = 1|H_1) = \frac{1}{3}Z_1, \quad \text{logit } P(A_2 = 1|H_2, A_1) = \frac{1}{3}Z_1 + \frac{1}{2}A_1.$$

The cumulative reward $Y$ is assumed to follow the nonlinear model:

$$\begin{aligned} Y = 1 + Z_1^2 + Z_2^2 + 2A_1\Big((Z_1+3)^2 + (Z_2+3)^2 + (Z_3+3)^3 - 30\Big) \\ + 2A_2\Big((Z_4+3)^2 + (Z_5+3)^2 + (Z_6+3)^2 + (Z_7+3)^2 - 40\Big) + \epsilon_Y, \end{aligned}$$

where $\epsilon_Y$ denotes random noise following the standard normal distribution. We note that, by design, the true significant variables for stage 1 and stage 2 are $(Z_1, Z_2, Z_3)$ and $(Z_4, Z_5, Z_6, Z_7)$, respectively.

In this study, we also run 500 simulation replicates with a training sample size of $N = 400$, and the performance is evaluated on an independent testing dataset with a sample size of 5,000 using the Monte Carlo method. All methods are implemented following the same description provided in Section 4 of the main paper. The results are summarized in Table S.1.

From the table, we first note that, similar to the second simulation setting in Section 4 of the main paper, A-learning, Q-learning, and dWOLS tend to perform worse compared to L1-MRL due to model misspecification. In contrast, L1-MRL and O-learning are machine learning-based algorithms and are, therefore, less sensitive to model misspecification, resulting in higher testing rewards. Compared to O-learning, L1-MRL shows better performance in terms of variable selection, as it can identify truly important variables with high probability while incurring a much lower number of false positives.

Table S.1: Summary of the testing reward, the number of false negatives (FN), false positives (FP), the false discovery rate (FDR), and the Jaccard index (JI) for the additional simulation setting.

| Method | Testing Reward | Stage 1 | | | | Stage 2 | | | |
|---|---|---|---|---|---|---|---|---|---|
| | | FN[2] | FP[2] | FDR | JI | FN[3] | FP[3] | FDR | JI |
| L1-MRL | 42.201 (1.940)[1] | 0.030 (0.171) | 2.288 (1.870) | 0.155 (0.124) | 0.584 | 0.086 (0.295) | 2.388 (1.774) | 0.165 (0.117) | 0.691 |
| A-learning | 38.297 (2.701) | 0.700 (0.596) | 0.000 (0.000) | 0.047 (0.040) | 0.634 | 1.834 (0.675) | 0.002 (0.045) | 0.122 (0.045) | 0.538 |
| O-learning | 43.855 (0.457) | 0.000 (0.000) | 4.020 (2.420) | 0.268 (0.161) | 0.226 | 0.000 (0.000) | 3.844 (2.347) | 0.256 (0.156) | 0.687 |
| Q-learning | 29.445 (5.470) | 2.370 (0.859) | 2.208 (2.397) | 0.305 (0.130) | 0.462 | 0.000 (0.000) | 5.644 (1.847) | 0.376 (0.123) | 0.534 |
| dWOLS | 17.744 (8.387) | 1.480 (1.229) | 5.468 (4.861) | 0.463 (0.259) | 0.258 | 1.815 (1.649) | 5.006 (4.494) | 0.455 (0.212) | 0.271 |

[1] Results are reported in *mean(standard deviation)* format.
[2] For stage 1, FN/FP denotes the number of estimated coefficients with absolute values greater than $10^{-6}$ among/outside $\{Z_1, Z_2, Z_3\}$.
[2] For stage 2, FN/FP denotes the number of estimated coefficients with absolute values greater than $10^{-6}$ among/outside $\{Z_4, Z_5, Z_6, Z_7\}$.

Additionally, L1-MRL achieves a higher Jaccard index, indicating more stable variable selection. Hence, the results demonstrate that L1-MRL still outperforms other methods when the important variables and the total number of important variables differ in each stage. In particular, the mean number of variables falsely identified as important among $(Z_8, ..., Z_{12}, X_1, X_2, X_3)$, i.e., the set of variables that are unimportant for both stages, is 0.530 and 1.422 (with standard deviations of 0.093 and 1.084) for each stage for L1-MRL, respectively. This suggests that L1-MRL maintains the oracle property and can select the union of variables that are important for at least one stage, which is also consistent with the conclusion of Theorem 1 in the main paper.

## S.2 Additional information for the real data application

### S.2.1 An illustrative example of treatment stage inference based on observed medication records

In this section, we provide a concrete example to illustrate how treatment stages are inferred from a patient's observed prescription records, as described in Section 5 of the main paper. The example is visualized in Figure S.1. In this example, we assume that six medication prescriptions are observed between 2008/01/01 and 2018/12/31 at consecutive time points $T1$ to $T6$, with prescribed medications denoted by $M1$ to $M6$. We further assume that the time gaps between $T1$ to $T6$ are 42, 210, 10, 12, and 250 days, respectively.

Following the description in the main paper, we begin the inference from time point $T1$, which is the first prescription since 2008/01/01. This also sets the initial time point of the first-stage treatment

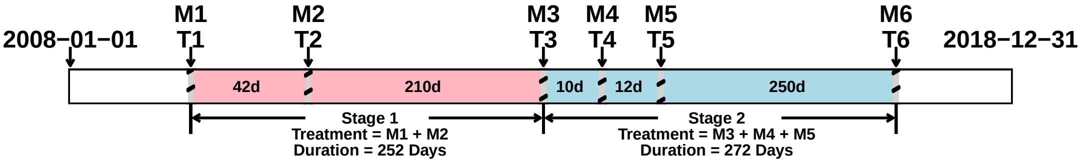


Figure S.1: An illustrative example of inferring treatment stages from the observed prescription records of a patient.

to be $T1$ and adds the medication $M1$ to the first-stage treatment. For the next time point $T2$, since $T2 - T1$ is 42 days and is less than the 180-day threshold, we extend the first-stage treatment to $T2$, add $M2$ to the first-stage treatment, and continue the search. As the third medication occurs at $T3$ and $T3 - T2$ is equal to 210 days, which is greater than the 180-day threshold, $T3$ will be identified as the initial time point of a different treatment stage according to the criterion. Consequently, we extend the first-stage treatment until $T3$ and terminate the inference for the first stage. This results in the first-stage treatment starting from time point $T1$, lasting for $42 + 210 = 252$ days till $T3$, with identified treatment $M1 + M2$.

For the second treatment stage, we repeat the previous procedures starting from $T3$. Since both $T4 - T3$ and $T5 - T4$ are less than 180 days, we extend the treatment twice until $T5$ and add $M4$ and $M5$ as treatments for the second stage, along with the initial medication $M3$. Finally, since $T6 - T5$ is greater than 180 days, we terminate the inference. Consequently, the second treatment stage starts at $T3$, lasting for $10 + 12 + 250 = 272$ days until $T6$, with the identified treatments $M3 + M4 + M5$. To summarize, according to the adopted criterion, we identify two treatment stages from Figure S.1: the first treatment stage (highlighted in red), starting from $T1$ and lasting for 252 days, with the identified treatments $M1+M2$, and the second treatment stage (highlighted in blue), starting from $T3$ and lasting for 272 days, with the identified treatments $M3 + M4 + M5$.

### S.2.2 Variable selection frequency table for the real data application

| Variable | L1-MRL | | A-learning | | Q-learning | | O-learning | | dWOLS | |
|---|---|---|---|---|---|---|---|---|---|---|
| | Stage 1 | Stage 2 | Stage 1 | Stage 2 | Stage 1 | Stage 2 | Stage 1 | Stage 2 | Stage 1 | Stage 2 |
| Age | 60 | 61 | 118 | 162 | 83 | 25 | 159 | 144 | 17 | 1 |
| Gender | 155 | 134 | 158 | 153 | 57 | 12 | 251 | 241 | 54 | 0 |
| Smoking | 79 | 76 | 88 | 177 | 77 | 29 | 153 | 164 | 13 | 0 |
| BMI | 292 | 174 | 213 | 149 | 101 | 17 | 5 | 73 | 82 | 0 |
| SBP | 153 | 109 | 96 | 141 | 47 | 22 | 159 | 128 | 8 | 0 |
| LDL | 50 | 50 | 70 | 206 | 56 | 27 | 208 | 58 | 21 | 0 |
| HDL | 114 | 84 | 107 | 158 | 67 | 6 | 175 | 143 | 96 | 0 |
| Triglyceride | 59 | 66 | 55 | 132 | 41 | 19 | 112 | 168 | 11 | 0 |
| HbA1c | 378 | 401 | 379 | 415 | 159 | 185 | 346 | 304 | 496 | 1 |

Table S.2: Variable selection frequency table for each method across 500 repeated analyses.
